# Defect-Mediated Catalysis for Low-Temperature Formation of Graphene-Based Materials

**DOI:** 10.1021/jacs.5c20150

**Published:** 2026-06-08

**Authors:** Mengxuan Zhang, Takeharu Yoshii, Qi Zhao, Yuichiro Hayasaka, Devis Di Tommaso, Hirotomo Nishihara

**Affiliations:** † Institute of Multidisciplinary Research for Advanced Materials, 13101 Tohoku University, 2-1-1 Katahira, Aoba-ku, Sendai, Miyagi 980-8577, Japan; ‡ School of Physical and Chemical Sciences, 4617 Queen Mary University of London, Mile End Road, London E1 4NS, U.K.; § The Electron Microscopy Centre, Tohoku University, 2-1-1 Katahira, Aoba, Sendai, Miyagi 980-8577, Japan; ∥ Advanced Institute for Materials Research (WPI-AIMR), Tohoku University, 2-1-1 Katahira, Aoba-ku, Sendai, Miyagi 980-8577, Japan

## Abstract

Achieving low-temperature graphene formation remains a major challenge in carbon materials chemistry. Here we reveal a defect-mediated catalytic mechanism in which dynamically generated oxygen vacancies on ceria (CeO_2_) activate acetylene (C_2_H_2_) and direct the structural evolution of carbon networks at remarkably low temperatures. The oxygen-vacancy–driven redox dynamics of CeO_2_ enables C_2_H_2_ decomposition to proceed at temperatures as low as 113 °C, initiating carbon nucleation and leading to graphene domain formation below 300 °C. The temperature-dependent evolution—from graphene quantum dots (GQDs, 300 °C) to aggregated graphene (450 °C) and porous graphene frameworks (600 °C)—illustrates a designable transition in carbon connectivity directed by defect chemistry. Mechanistic studies combining *in situ* spectroscopy, thermogravimetry, and density functional theory reveal that the reaction follows a temperature-dependent transition from a radical to a carbene pathway, governed by the oxygen-vacancy chemistry of CeO_2_. Together, these results define a defect-mediated catalytic paradigm that couples oxide redox dynamics with carbon dimensionality control, offering a general principle for low-temperature formation of graphene-based sp^2^ carbon materials.

## Graphical abstract

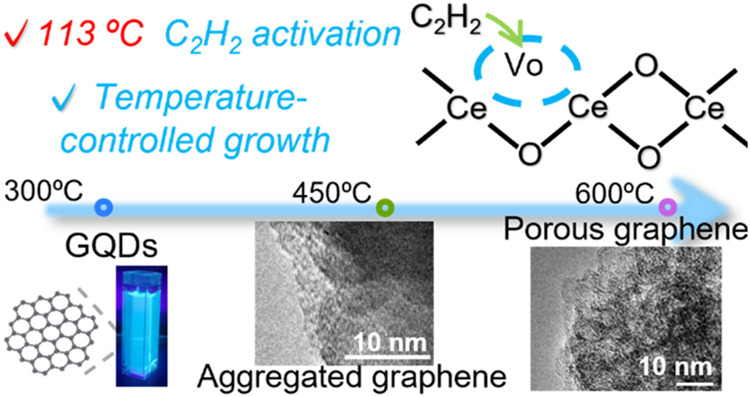

## 1 Introduction

Graphene, with single-layer hexagonal lattice arranged in sp^2^ hybridized carbon atoms, has received much attention from various fields since it was isolated from graphite in 2004.^1,2^ Graphene can be synthesized via two principal approaches: a top-down route, exemplified by chemical exfoliation of graphite, and a bottom-up route. Among these, the bottom-up approach offers a promising and scalable method for producing high-quality graphene, most notably chemical vapor deposition (CVD).^3,4^ In particular, template-assisted CVD has emerged as a powerful strategy, enabling not only the synthesis of two-dimensional graphene sheets but also the fabrication of three-dimensional (3D) graphene architectures.^5^ Experimentally, 3D graphene can be synthesized via CVD on both metal and nonmetal templates. While porous transition metals such as Cu and Ni are effective CVD substrates for large-area graphene growth, their thermal instability confines the pore size to the micrometer range.^5,6^ In contrast, nonmetal templates are more suitable for fabricating 3D graphene with enriched pore architecture and diverse morphologies. Ordered microporous carbon materials featuring interconnected 1.2 nm nanopores and large surface areas have been synthesized using zeolite templates via CVD.^7,8^ More recently, elastic graphene mesosponge (GMS) with single-layer graphene walls have been developed using alumina (Al_2_O_3_),^9,10^ magnesium oxide (MgO),^11^ calcium oxide (CaO),^12^ and silica (SiO_2_)^13^ as templates. This synthesis typically involves CVD with mechane (CH_4_) as the carbon precursor, forming a conformal monolayer carbon coating on the template surface. Removal of the oxide template by chemical etching yields a carbon mesosponge (CMS), which upon annealing at 1800 °C undergoes a graphene zipping reaction to form a fully interconnected GMS framework.^14^

Although considerable efforts have been devoted to developing CVD processes on nonmetal templates, several challenges remain. While CH_4_ is widely employed as a carbon precursor for synthesizing low-defect graphene like GMS, its high C–H dissociation energy demands high reaction temperatures, typically exceeding 900 °C.^15,16^ The high reaction temperature restricts the choice of templates, as only those capable of retaining both surface area and crystal structure above 900 °C can be used. Furthermore, once CH_4_ decomposition begins, graphene growth proceeds rapidly in an epitaxial manner, often yielding single-layer graphene within minutes.^17,18^ This rapid growth behavior hampers precise control over the size of graphene, thereby limiting the applicability of the CVD method for producing graphene materials with diverse structures. This limitation highlights the need for a controllable CVD strategy, particularly for the synthesis of graphene quantum dots (GQDs), which have attracted growing interest because of their tunable bandgap, high surface area, and abundant surface functional groups. To reduce the growth temperature, recent efforts in low-temperature CVD have focused on the application of external energy inputs (e.g., plasma or microwave^19,20^ ) or the selection of carbon precursors with lower decomposition temperatures.^21–24^ Among these, gaseous precursors such as acetylene (C_2_H_2_) are advantageous due to their relatively low activation energy (∼500–600 °C) compared with CH_4_.^25,26^

For the structural control of graphene during CVD, the choice of template is critically important. Recently, a defect–dominated catalytic mechanism on metal-oxide templates at high temperatures has been identified. Specifically, oxygen vacancies (V_O_) on oxides such as MgO and Al_2_O_3_ have been recognized as key active sites for the catalytic decomposition of CH_4_, thereby facilitating carbon deposition during CVD growth.^11^ While these findings highlight the critical role of surface defects, the correlation between V_O_-driven catalysis and the structural evolution of graphene remains largely unclear. In particular, how V_O_-mediated redox dynamics influence hydrocarbon activation and carbon nucleation under low-temperature conditions has yet to be clarified.

To gain further insight into this mechanism, CeO_2_ was selected as a representative reducible oxide template, given its ability to release lattice oxygen and stabilize oxygen vacancies even under mild conditions via the reversible Ce^4+^/Ce^3+^ redox transition.^27,28^ Besides, ceria (CeO_2_) has also been reported to catalyze C_2_H_2_ conversion and decomposition. It is indicated that C_2_H_2_ can adsorb on CeO_2_ in multiple configurations and form dissociative C_2_H_*x*_ intermediates,^29,30^ suggesting strong dependence on the vacancy-rich surface state. Complementary theoretical and experimental work further proposed that Vo-associated Ce/O site pairs can lower key activation barriers and modulate reaction pathways,^31,32^ underscoring the mechanistic importance of oxygen vacancies in CeO_2_-mediated hydrocarbon activation. However, most previous studies have mainly focused on the activation and transformation of small hydrocarbon molecules, whereas the subsequent carbon-formation processes toward extended sp^2^ carbon structures have received much less attention.

In this study, we demonstrate a defect-mediated catalytic strategy for the controllable synthesis of graphene-based materials via low-temperature CVD using CeO_2_ as a reducible template and C_2_H_2_ as a carbon source (Figure 1). Multiple *in situ*characterizations revealed that C_2_H_2_ begins to decompose at a remarkably low temperature of 113 °C on CeO_2_ through oxygen-vacancy-driven redox dynamics, leading to the formation of sp^2^ carbon structure below 300 °C. Building on these insights, we systematically varied the reaction temperature and successfully obtained distinct graphene-based structures, including GQDs with blue fluorescence at 300 °C and porous graphene with a high surface area at 600 °C. This study introduces a new catalytic design concept that couples defect state dynamics with carbon network formation and transforms the way low-temperature growth of graphene-based carbon materials can be achieved. More broadly, it establishes a general paradigm in which redox active oxide templates guide carbon dimensionality through defect-mediated reaction pathways. This concept transforms catalytic carbon growth from a temperature-controlled process into a defect-governed design principle, redefining how oxide templates can be utilized for carbon material synthesis.

**Figure 1:**
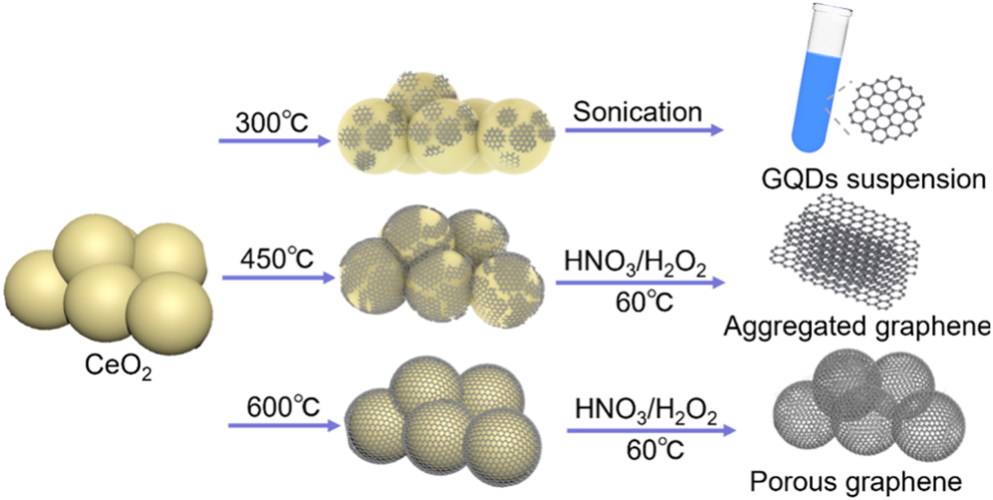
Schematic of controllable growth of graphene-based materials.

## 2 Experimental Section

### 2.1 Analysis of C_2_H_2_ Decomposition Behavior on CeO_2_

#### 2.1.1 *In Situ* CVD-TG Measurement

Starting temperature of catalytic C_2_H_2_ deposition on the surface of CeO_2_ (CeO_2_–HS, 99.9%, DAIICHI KIGENSO KAGAKU KOGYO CO., LTD, BET specific surface area: 121 m^2^ g^–1^) was investigated by *in situ* CVD-Thermogravimetry (TG). First, CeO_2_ was placed into the TG chamber (STA2500 Regulus; NETZSCH Japan), then a mixed gas (20 mL min^–1^ C_2_H_2_ + 80 mL min^–1^ Ar) was introduced. The weight change was monitored with a heating rate of 1 °C min^–1^ until 300 °C. For comparison, the same *in situ* CVD-TG was also applied to MgO (US Research Nanomaterials, 30 nm, BET specific surface area: 61 m^2^ g^–1^).

*In situ* CVD-TG was also applied to investigate the C_2_H_2_–CVD process at a constant temperature. The TG chamber with CeO_2_ or MgO was heated to a specified temperature at 10 °C min^–1^ under a steady Ar flow (100 mL min^–1^). After being maintained 30 min, the Ar gas was switched to a mixed gas (20 mL min^–1^ C_2_H_2_ + 80 mL min^–1^ Ar) and the reaction was continued for 2 h. Here the weight increase from C_2_H_2_ decomposition was converted into the average stacking number of graphene layers (The detailed calculation method is described elsewhere.^11^). The activation energy (*E*_a_) for C_2_H_2_ decomposition was calculated by Arrhenius equation.^13^

#### 2.1.2 *In Situ* DRIFTS and *In Situ* Raman

The surface change of CeO_2_ during C_2_H_2_–CVD process was examined by *in situ* diffuse reflectance infrared Fourier-transform spectroscopy (*in situ* DRIFTS) mode using an S.T. Japan Inc. Heat Chamber Type-1000 °C/DiffusIR mounted on a Shimadzu IRSpirit-X with BaF_2_ window. First, the chamber containing CeO_2_ was heated up to a target temperature under Ar flow (200 mL min^–1^) and maintained for 30 min. Then, a mixed gas (2 mL min^–1^ C_2_H_2_ + 198 mL min^–1^ Ar) was introduced and maintained for 30 min with continuous IR recording. The obtained samples were analyzed by X-ray photoelectron spectroscopy (XPS; JEOL, JPS-9200) with Al Kα radiation without exposure to air. In addition, Raman measurement (LabRAM HR800, HORIBA Jobin Yvon) was performed for the air-exposed samples using the 532.2 nm line.

*In situ* Raman spectroscopy was also conducted following the same procedure as that used for *in situ* DRIFTS, except that the BaF_2_ window was replaced with a quartz window, and spectra were acquired using a Raman spectrometer (DXR3 Flex, Thermo Fisher Scientific).

#### 2.1.3 *In Situ* CVD-MS

The gas emission during C_2_H_2_–CVD process was examined by *in situ* CVD-mass spectrometry (MS, BELMASS II, MicrotracBEL Corp.). First, CeO_2_ (∼1 g) was placed into tube furnace, and helium (He) with a flow rate of 200 mL min^–1^ was introduced. Then, the sample was heated to a specified temperature with a heating rate of 10 °C min^–1^. After all signals stabilized, the He flow was exchanged to a mixed gas flow (2 mL min^–1^ C_2_H_2_ + 198 mL min^–1^ He). The intensity of generated gas was converted into molecular amounts using calibration curves obtained from standard gases.

### 2.2 Theoretical Calculations

All spin-polarized DFT calculations were conducted using the Vienna *ab initio* simulation package (VASP).^33^ Electronic exchange and correlation were treated within the generalized gradient approximation (GGA) by using the Perdew Burke Ernzerhof (PBE) function, together with the Grimme’s-D3 dispersion correction, to provide a more accurate description of the ion-induced dipole interaction.^34,35^ Valence electrons were described by a plane wave basis set with an energy cutoff (*E*_cut_) of 450 eV to optimize the intermediates on the reaction energy profile. In this study, the DFT and DFT + *U* methods with *U*_eff_ = 4.5 eV were performed to accurately correct the strong on-site Coulomb repulsion of Ce 4f states on reduced ceria surfaces. The CeO_2_(110) surface was modeled as a periodic slab with 3 × 3 super cell and the vacuum between neighboring slabs was set to 15 Å. All the atoms were allowed to relax during optimization, the energy convergence criterion was set 10^–5^ eV and the force threshold was set to 0.01 eV/Å. k-point mesh was set to 3 × 3 × 3 in Brillouin zone. Ab initio molecular dynamics (AIMD) simulations were performed in the canonical constant-volume, constant-temperature (NVT) ensemble. The system was gradually heated from 400 to 500 K for 2 fs with a fixed-shape volume fluctuation. Each simulation was run for 10,000 steps with a time step of 2 fs, corresponding to a total simulation time of 20 ps.

Adsorption energies were computed using the following expression

$$E_{\mathrm{ads}}=E\left(\mathrm{adsorbate}+\mathrm{slab}\right)-E\left(\mathrm{adsorbate}\right)-E\left(\mathrm{slab}\right)$$

where the first term is energy of the optimized surface slab with the adsorbate, the second term is the energy of the isolated optimized adsorbate molecule, and the third term is the energy of the optimized bare surface slab.

### 2.3 Preparation of C/CeO_2_ Composite and Carbon Samples

CeO_2_ (1 g) was put into the vertical furnace and Ar was introduced as a flow rate of 225 mL min^–1^. Then, the furnace was heated to a target temperature at 10 °C min^–1^. After being maintained for 30 min, the Ar gas was switched to a mixed gas (C_2_H_2_ 45 mL min^–1^ + Ar 180 mL min^–1^) for 30 min. Then, the pure Ar was flowed again until the furnace was cooled down to room temperature. Afterward, a mixed gas with 3.5% oxygen (air 100 mL min^–1^ + Ar 477 mL min^–1^) was introduced to the furnace and maintained 6 h to prevent a rapid exothermic reaction between CeO_2_ and air, which could damage the carbon coating. The obtained C/CeO_2_ samples are denoted as C/CeO_2_-*x*, where *x* represents the reaction temperature.

For the preparation of GQD suspension, C/CeO_2_-300 was dispersed in ethanol and sonicated for 1 h. After filtration, the GQD suspensions were obtained. CeO_2_ template for C/CeO_2_-450 and C/CeO_2_-600 was removed by chemical etching. Specifically, the obtained carbon coated CeO_2_ was added into HNO_3_ (8M, Wako Pure Chemical Industries, Japan) and heated to 60 °C, then H_2_O_2_ solution (30 wt %, Wako Pure Chemical Industries, Japan) was added slowly and treated for 2 h. The remaining materials were collected by filtration and dried under vacuum at 60 °C to obtain C-450 and C-600, respectively.

### 2.4 Preparation of Morphology-Controlled CeO_2_ Templates (CeO_2_-Rod and CeO_2_-Octa)

#### 2.4.1 Preparation of CeO_2_ Nanorods

NaOH solution (56 mL, 6.87 M) was added dropwise into an aqueous solution of Ce­(NO_3_)_3_·6H_2_O (8 mL, 0.4 M), then the slurry was stirred for 30 min continually. The mixed solution was transferred into a Teflon-lined stainless-steel autoclave and hydrothermally treated at 100 °C for 24 h to obtain CeO_2_ nanorods (CeO_2_-rod). The obtained materials were separated and washed by centrifugation with deionized water and ethanol several times, then dried at 80 °C for 8 h. After CVD at 600 °C, the CeO_2_-rod template was removed using the same etching procedure described above, and the remaining carbon product was collected and denoted as C-rod.

#### 2.4.2 Preparation of CeO_2_ Octahedrons

Na_3_PO_4_ solution (56 mL, 0.66 mM) was added dropwise into an aqueous solution of Ce­(NO_3_)_3_·6H_2_O (8 mL, 0.4 M), and the mixture was continually stirred for 30 min. The mixed solution was transferred into a Teflon-lined stainless-steel autoclave and hydrothermally treated at 170 °C for 10 h. Then the obtained materials were separated and washed by centrifugation with deionized water and ethanol several times, then dried at 80 °C for 8 h to obtain CeO_2_ octahedron (CeO_2_-octa).

### 2.5 Characterization

The crystallinity of obtained samples was characterized by powder X-ray diffraction (XRD) analysis (MiniFlex600, Rigaku Co.) with Cu Kα radiation generated at 40 kV and 15 mA. The amount of carbon deposited on C/CeO_2_ was determined by TG analysis (TGA-50, SHIMADZU). The sample was heated from room temperature to 900 °C at a rate of 10 °C min^–1^ under an air flow. Nanostructures of the samples were observed by transmission electron microscope (TEM; JEM-2010, JEOL Ltd.), and selected-area electron diffraction (SAED) patterns were collected on the same instrument at an acceleration voltage of 100 kV. GQD samples were characterized by scanning transmission electron microscopy (STEM) and high-resolution TEM (HRTEM) using a Titan G2 60–300 double Cs-corrected microscope (FEI) equipped with an energy-dispersive X-ray spectrometer (Super-X) at acceleration voltages of 60 kV and 200 kV, respectively. The specimens were supported on super high-resolution carbon films (SHR-C075, Nisshin EM Co., Ltd.). Ultraviolet–visible (UV–vis) absorption spectrum of GQD suspension was recorded (V-770TWK, JASCO) with the wavelength ranging from 200 to 800 nm. Photoluminescence (PL) emission spectra were measured by FP8500, JASCO. The specific surface area (SSA) was characterized by N_2_ adsorption/desorption measurements at −196 °C using multipoint Brunauer-Emmett-Teller (BET) method. XPS and Raman analyses of the carbon materials were performed using the same methods as those described above for the samples after the *in situ* DRIFTS measurements. The amount of edge sites was estimated by an advanced temperature-programmed desorption (TPD) analysis up to 1800 °C.^36–38^ The electric conductivity was measured by a two-probe method while applying pressure to the powdery sample up to 50 MPa (HZ-7000, Meiden Hokuto Co.).^10^

## 3 Results and Discussion

### 3.1 Reaction of C_2_H_2_ Decomposition on CeO_2_

To establish the intrinsic catalytic capability of CeO_2_ for low-temperature hydrocarbon activation, we first investigated the decomposition behavior of C_2_H_2_. CeO_2_ nanoparticles exhibit a typical fluorite crystal structure with an average diameter of approximately 10 nm (Figure S1). To determine the onset temperature of C_2_H_2_ decomposition on CeO_2_, we conducted *in situ* CVD-TG measurements under flowing C_2_H_2_ with a heating rate of 1 °C min^–1^ from room temperature to 300 °C. As shown in Figure 2a, the weight of CeO_2_ initially decreases due to desorption of physically adsorbed H_2_O, and then starts to increase at 113 °C, indicating the onset of C_2_H_2_ decomposition to form a C/CeO_2_ composite. In comparison, MgO, which serves as a conventional catalytic template for 3D graphene synthesis, also exhibits an initial weight loss, which is mainly attributed to the desorption of preadsorbed H_2_O and CO_2_-derived surface species (Figure S2a). However, the subsequent weight increase is observed starting at 317 °C with H_2_ evolution (Figure S2b), a temperature significantly higher than that for CeO_2_. The significantly lower onset temperature for CeO_2_ suggests superior catalytic activity for C_2_H_2_ decomposition compared to MgO.

**Figure 2:**
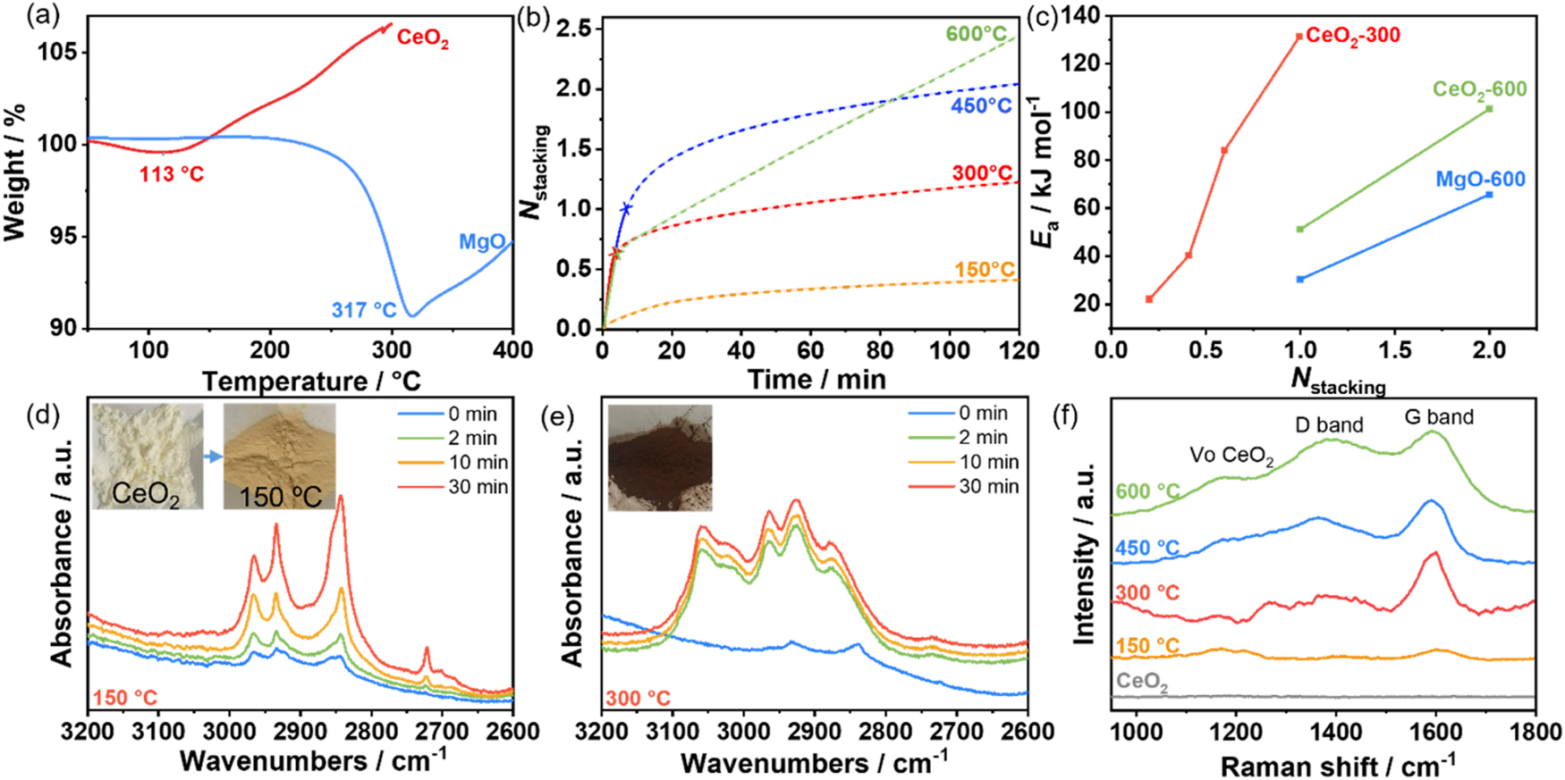
(a) Weight change of CeO_2_ and MgO under a mixed gas flow of C_2_H_2_ (20 vol %) and Ar monitored by TG. (b) Weight changes of CeO_2_ during C_2_H_2_–CVD under a mixed gas flow of C_2_H_2_ (20 vol %) and Ar at different temperatures monitored by TG. C_2_H_2_ was introduced to the reactor at 0 min. The solid line represents stage 1, while the dashed line corresponds to stage 2 at the reaction temperature. The “×” symbol indicates the transition point between the two stages. (c) Activation energy on CeO_2_ and MgO during C_2_H_2_–CVD at 300 and 600 °C. *In situ* DRIFTS on CeO_2_ during C_2_H_2_–CVD at (d) 150 °C and (e) 300 °C. The insets in (d, e) are sample images of C/CeO_2_ after DRIFTS. (f) Raman spectra of C/CeO_2_ after DRIFTS at different reaction temperatures.

The reaction kinetics were further analyzed by monitoring the weight change of CeO_2_ upon C_2_H_2_ introduction at various temperatures using *in situ* CVD-TG. The weight gain was converted to the number of graphene layers (*N*_stacking_) based on the specific surface area (SSA) of CeO_2_,^11^ as shown in Figures 2b and S3a. A noticeable weight increase at 150 °C confirms that C_2_H_2_ decomposition occurs even at such a low temperature. Interestingly, a two-stage growth behavior was consistently observed from 200 °C to 600 °C, and the characteristic 600 °C profile was reproducible in independent repeats (Figure S3b). In stage 1, *N*_stacking_ increases rapidly, followed by a significant slowdown in stage 2, where growth proceeds at a nearly constant rate. Here a straight line was fitted to the initial 0–3 min region of the *N*_stacking_ curve; the point where the growth rate falls below this line defines the end of stage 1, marked by a “×” symbol. *N*_stacking_ in stage 1 increases with temperature, approaching ∼1 layer at 450–500 °C, indicative of monolayer graphene coating, but decreases to ∼0.7 layer at 600 °C (Figure S3c). The rapid initial growth in stage 1 is attributed to the catalytic activity of CeO_2_. The decrease in *N*_stacking_ in stage 1 at over 550 °C might be caused by the decrease in the number of active sites available for C_2_H_2_ adsorption at a high temperature, which will be discussed in the later section. Meanwhile, the slope of stage 2 defined at 60 min increases with temperature, reflecting enhanced carbon deposition (Figure S3d).

For comparison, the CVD-TG curves measured on MgO under continuous introduction of C_2_H_2_ at 600 °C was observed (Figure S3e). C_2_H_2_ decomposition on MgO shows a similar two-stage process as CeO_2_, but with a faster growth rate. It shows an extremely rapid growth rate at the beginning until *N*_stacking_ ≈ 2, followed by a slower growth rate. Such behavior suggests achieving a uniform coating of MgO with a single-graphene layer via C_2_H_2_–CVD is difficult. Compared with MgO, CeO_2_ exhibits a relatively slow growth rate when *N*_stacking_ is above 0.7, showing the potential to control the decomposition rate on CeO_2_ for the generation of single-layer carbon coating.

Remarkably, the previously studied CH_4_-to-C reaction on MgO undergoes an induction period during the initial 3 min, corresponding to the activation process (Figure S4a).^11^ In this period, CH_4_ removes O atoms from the MgO surface as CO to form Vo. Also, the CH_4_-to-C conversion on CeO_2_ shows a decreasing trend during the initial 20 min (Figure S4b), which corresponds to a similar activation process involving the formation of V_O_. This decrease is attributed to the excellent oxygen-releasing capacity of CeO_2_, resulting in a pronounced weight loss. In comparison, C_2_H_2_-to-C process did not exhibit a visible induction period but a rapid increase (see Figure 2b), suggesting a faster and different activation process of C_2_H_2_ decomposition from the case for CH_4_.

To further investigate the two-step reaction mechanism during the progression of the C_2_H_2_–CVD process above 300 °C, we calculated the time-dependent activation energy (*E*_a_) by Arrhenius plots based on the carbon deposition rate obtained from the CVD-TG curves at approximately 300 and 600 °C (Figures S5 and S6). The results are summarized in Figure 2c. For comparison, *E*_a_ for MgO at 600 °C is also included. At 300 °C, *E*_a_ on CeO_2_ is calculated to be as low as 22 kJ mol^–1^ at the initial stage, and it gradually increases as carbon deposition proceeds, reaching 132 kJ mol^–1^ when *N*_stacking_ reaches 1. In contrast, as shown in Figure 2a, carbon deposition on MgO is barely observed at 300 °C, making it difficult to determine a meaningful value of *E*_a_ at this temperature. The low initial *E*_a_ on CeO_2_ confirms its excellent catalytic ability and highlights its superior potential for low-temperature graphene growth compared to MgO. Interestingly, the *E*_a_ values of CeO_2_ and MgO are reversed at 600 °C: for the formation of the first graphene layer, *E*_a_ is 51.2 kJ mol^–1^ on CeO_2_ and 30.4 kJ mol^–1^ on MgO, while for the second layer, the values are 101.3 kJ mol^–1^ on CeO_2_ and 65.7 kJ mol^–1^ for MgO. The slower graphene growth rate on CeO_2_ at 600 °C can be attributed to its unique surface properties, which moderate the decomposition of C_2_H_2_ and prevent uncontrolled carbon accumulation. This contrasts with the rapid, less controllable growth observed on MgO. The exceptionally low activation energy at 300 °C and the suppressed reaction rate at elevated temperatures demonstrate that CeO_2_ offers not only a remarkably low-temperature onset for graphene nucleation but also superior tunability over the kinetics of growth, exhibiting a high potential for the precise synthesis of structurally controlled graphene-based materials.

To elucidate the graphene formation process, surface changes of CeO_2_ during CVD at various temperatures were continuously monitored using *in situ* DRIFTS under a flow of C_2_H_2_. At 150 °C (Figure 2d), peaks at 2966 and 2940 cm^–1^ are assigned to the C–H stretching of sp^3^-hybridized carbon derived from C_2_H_2_ decomposition. Additionally, the bands at 2840 and 2720 cm^–1^ can be assigned to symmetric C–H stretching and Fermi resonance modes characteristic of aldehyde groups (−CHO), respectively. Also, Figure S7a shows the peaks at 1360 and 1430 cm^–1^ which are attributed to C = O stretching, further confirming the presence of −CHO species. The gradual increase in peak intensity and the slight color change from pale yellow to light brown in 30 min indicate a slow decomposition rate (see Figure 2d), and the absence of sp^2^-related signals suggests that graphene sheets are not formed at this temperature. Note that weak bands around 2800–3000 cm^–1^ observed at *t* = 0 min are attributed to H_2_O/CO_2_-related surface adsorbates on CeO_2_ (Figure S7a). After removing the preadsorbed species by heating CeO_2_ in Ar at 600 °C (Figure S7b) and subsequently cooling to 150 °C, the introduction of C_2_H_2_ still gives rise to the same C–H and −CHO related bands (Figure S7c), supporting that these features originate from C_2_H_2_ conversion rather than residual adsorbates.

At 300 °C, the spectral intensity remains largely unchanged over time (Figure 2e), consistent with the rapid weight gain at the initial stage, observed in the CVD-TG curves (see Figure 2b). The −CHO-related peaks are still detectable but are significantly weaker compared to those at 150 °C. Meanwhile, new peaks emerge at 3060 and 3030 cm^–1^, attributed to the C–H stretching of sp^2^-hybridized carbon, indicating the onset of graphene domain formation. At 450 °C (Figure S7d), both sp^3^ and sp^2^ C–H stretching peaks are present, and the sample exhibits a more pronounced color change to black, suggesting more extensive carbon growth. At 600 °C (Figure S7e), only a transient peak at 3050 cm^–1^ (C=C) is observed during the first 2 min, after which all peaks disappeared. This behavior reflects the rapid surface coverage by carbon species, which likely renders the CeO_2_ surface IR-inactive.

The quality of the deposited carbon materials was further evaluated by Raman spectroscopy, as shown in Figure 2f. No detectable peaks can be observed after reacted at 150 °C, but other samples exhibit characteristic peaks at around 1317 and 1605 cm^–1^, corresponding to the D and G bands of carbon materials, respectively. Notably, the raw spectrum at 300 °C shows an upward baseline trend (Figure S7f), likely attributable to the fluorescence effect of the formed GQDs on CeO_2_, which will be discussed in the later section. In addition, a weak band appears around 1160 cm^–1^, which is associated with defect-related vibrations in CeO_2_, suggesting the formation of oxygen vacancies (V_O_) during C_2_H_2_ decomposition.^39^ Taken together, it is indicated that C_2_H_2_ begins to decompose on CeO_2_ at 150 °C, while significant graphene formation occurs at temperatures above 300 °C.

### 3.2 Mechanism of Catalytic C_2_H_2_ Decomposition on CeO_2_

It is widely acknowledged that the decomposition of CH_4_, a prototypical saturated hydrocarbon, proceeds via a radical mechanism under noncatalytic gas-phase conditions.^40^ In contrast, the activation of C_2_H_2_ can proceed through two distinct pathways: radical formation (reaction 1) and carbene formation (reaction 2).

1 $$C_{2}H_{2}\to {C_{2}H}^{•}+H^{•}$$

2 $$C_{2}H_{2}\to :\mathrm{CC}H_{2}$$

Numerous studies on the noncatalytic decomposition of C_2_H_2_ have suggested that the molecule preferentially undergoes carbene formation, rather than a free-radical pathway, since carbene formation (186 kJ mol^–1^) is energetically more favorable than radical formation (500 kJ mol^–1^).^41–44^ Here, to further clarify the initial activation process for C_2_H_2_ decomposition on CeO_2_, density functional theory (DFT) calculations were carried out on CeO_2_ (110) surface (Figure S8). We first investigated the C_2_H_2_ adsorption behavior using DFT-based ab initio molecular dynamics (AIMD) simulations. The carbon atoms in C_2_H_2_ were found to preferentially adsorb onto surface oxygen atoms of CeO_2_, leading to the formation of C–O bonds (Video S1). Based on these observations, static DFT calculations were conducted to explore the subsequent activation pathways. The results revealed that, after the weak physisorption of C_2_H_2_ on the CeO_2_ (110) surface (*E*_ads_= −0.49 eV) to form C_2_H_2_* (where * denotes an adsorbed species), the homolytic cleavage of a C–H bond to produce C_2_H* and H* is energetically favored, with an associated energy change of −2.85 eV. In contrast, self-rearrangement to form CCH_2_* results in a less exothermic energy change of −1.09 eV (Figure 3a). These results suggest that the initial activation of C_2_H_2_ on CeO_2_ is more likely to proceed via the radical formation pathway (reaction 1), although both pathways are exothermic and thermodynamically favorable. Following dissociative adsorption, the surface-bound C_2_H* undergoes further decomposition into C* and CH* with an activation barrier of 0.90 eV. The resulting CH* can subsequently participate in a coupling reaction with a migrated H* (migration barrier *E*_m_ = 0.2 eV), forming CH_2_* adsorbed onto surface oxygen atoms. Subsequently, CH_2_* and C* are capable of desorbing from the CeO_2_ surface through lattice oxygen abstraction, forming gaseous HCHO and CO, with the desorption energies of −1.0 and 0.07 eV, respectively. Thus, this sequence of reactions ultimately results in the formation of oxygen vacancies (V_O_) on the CeO_2_ surface.

**Figure 3:**
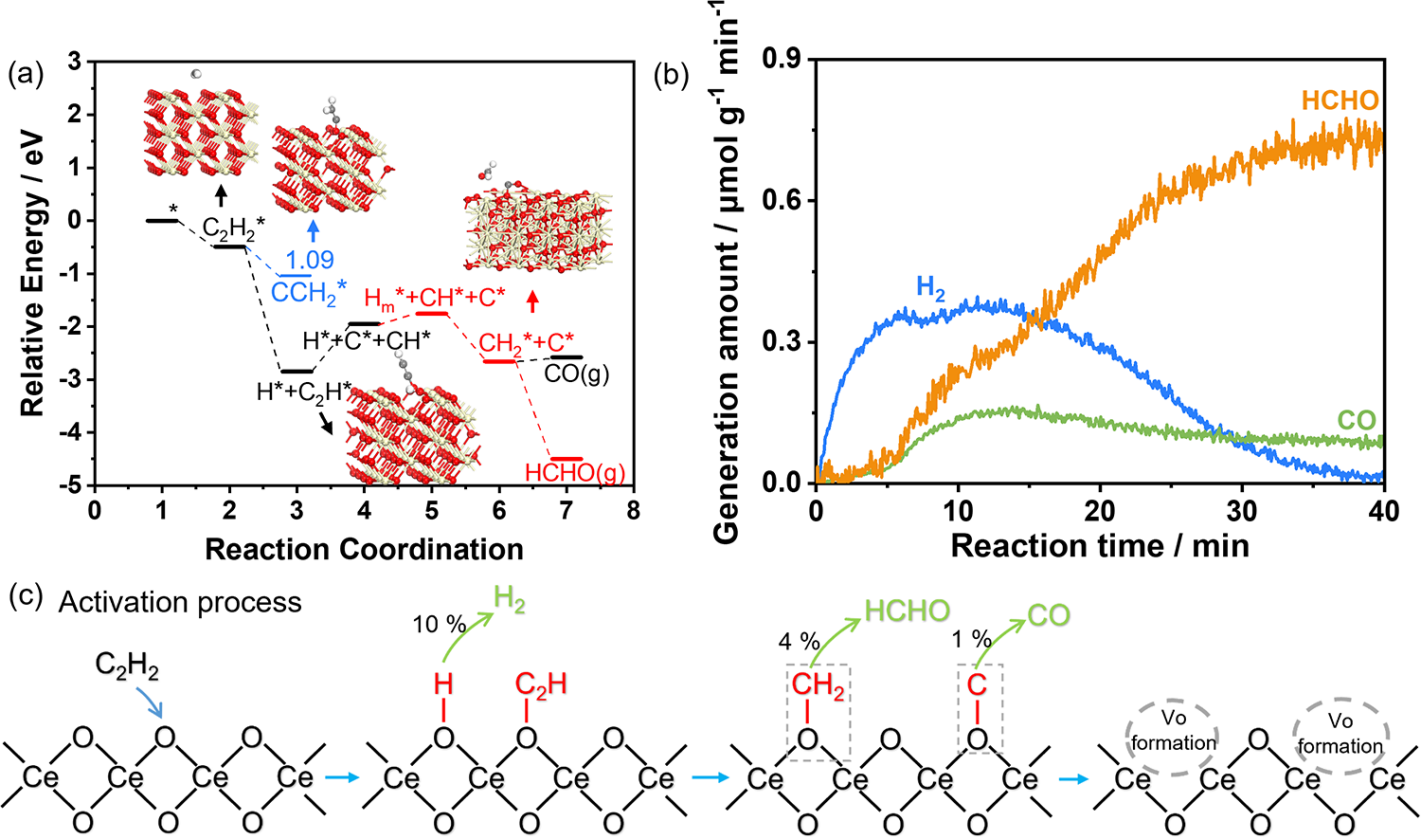
(a) Energy profiles of the C_2_H_2_ conversion on CeO_2_(110) surface. The energy of CeO_2_ surface before adsorption is set as 0 eV. The asterisk (*) denotes a molecule adsorbed on the CeO_2_ surface. (b) Gas emission during catalytic C_2_H_2_ decomposition on CeO_2_ at 150 °C. C_2_H_2_ (1 vol % in He) was introduced to the reactor at 0 min. (c) Schematic for the mechanism of C_2_H_2_ activation and decomposition on CeO_2_.

To experimentally validate the proposed C_2_H_2_ activation process, the gaseous products generated during C_2_H_2_ decomposition at 150 °C were collected and analyzed by CVD-mass spectrometry (MS). As shown in Figure 3b, gaseous species with molecular weights of 2 and 28 were detected, corresponding to the formation of H_2_ and CO, respectively. In addition, a signal at *m*/*z* = 30 is attributed to the generation of formaldehyde (HCHO), consistent with the DFT-predicted reaction pathways. The formation of surface-bound CH_2_* intermediates is also supported by the presence of −CHO groups observed via *in situ* DRIFTS (see Figure 2d). *In situ* Raman spectroscopy further corroborates concurrent defect formation of CeO_2_ under C_2_H_2_ at 150 °C. No new bands above 800 cm^–1^ appear upon heating in Ar at 150 °C (Figure S9a), whereas introducing C_2_H_2_ generates a weak feature at ∼935 cm^–1^ (Figure S9b), assignable to a defect-activated second-order Raman feature of CeO_2_. Moreover, a gradual red shift of the F_2g_ mode is observed during the CVD period in the presence of C_2_H_2_ but not under Ar heating (Figure S9c,d), consistent with defect (Vo/Ce^3+^)-associated lattice perturbation.^39,45^ These results provide evidence that C_2_H_2_ decomposition on CeO_2_ proceeds via a radical mechanism, in agreement with the simulation study, unlike the conventional C_2_H_2_ decomposition in the absence of a catalyst. Accordingly, the proposed initial reaction sequence is summarized in Figure 3c. C_2_H_2_ initially undergoes dissociative adsorption to form surface-bound C_2_H and H species, of which some H recombines to release H_2_ (10%). Meanwhile, the C–C bond in the surface-bound C_2_H species cleaves and reacts with migrated surface H atoms, forming surface-bound CO and HCHO-like intermediates. These species desorb in the form of gaseous CO and HCHO, thereby generating V_O_ on the CeO_2_ surface. Note that the amounts of HCHO and CO shown in Figure 3b are significantly lower than the amount of deposited carbon on CeO_2_ (Figure S10), with only ∼4% and ∼1% released as HCHO and CO, respectively. This indicates that only a fraction of the adsorbed species on CeO_2_ desorbs as gaseous products. Most of the carbon-containing species remain adsorbed on the surface and show a tendency for further reaction. However, the light sample color and the absence of sp^2^ C–H signals in *in situ* DRIFTS spectra (see Figure 2d) suggest the limited reaction rate at 150 °C. It is noteworthy that although C_2_H_2_ exhibits a similar spontaneous adsorption behavior on MgO as on CeO_2_, the subsequent decomposition on MgO favors the formation of CCH_2_* rather than C_2_H*, suggesting a potentially different decomposition pathway on MgO compared to CeO_2_ (Figure S11).

Subsequently, to elucidate the reaction mechanism at higher temperatures, gaseous products evolved during catalytic C_2_H_2_ decomposition over the temperature range of 300–600 °C were collected and analyzed (Figures 4a–d and S12a). The immediate evolution of H_2_ upon C_2_H_2_ introduction is attributed to the accelerated decomposition kinetics at higher temperature (Figure 4a). In parallel, the production of CO increases with temperature (Figure 4b), reflecting enhanced oxygen release from the CeO_2_ surface (see Figure 3c). Notably, no HCHO species were detected when the reaction temperature exceeded 300 °C, suggesting that HCHO decomposes into H_2_ and CO at elevated temperatures due to its thermal instability. In addition to CO, CO_2_ was also detected (Figure S12a); however, significant desorption was observed only at 600 °C. This observation is rationalized by the reoxidation of desorbed CO via reaction with surface oxygen atoms, yielding CO_2_ through an exothermic pathway (*E*_a_ = −2.8 eV; Figure S12b). Given the relatively high desorption energy of CO_2_ (+0.7 eV), its release becomes significant only at 600 °C. Importantly, since CeO_2_ serves as the sole oxygen donor in the system, the formation of both CO and CO_2_ is intrinsically linked to the generation of V_O_, which plays a critical role in governing the unique graphene growth behavior observed.

**Figure 4:**
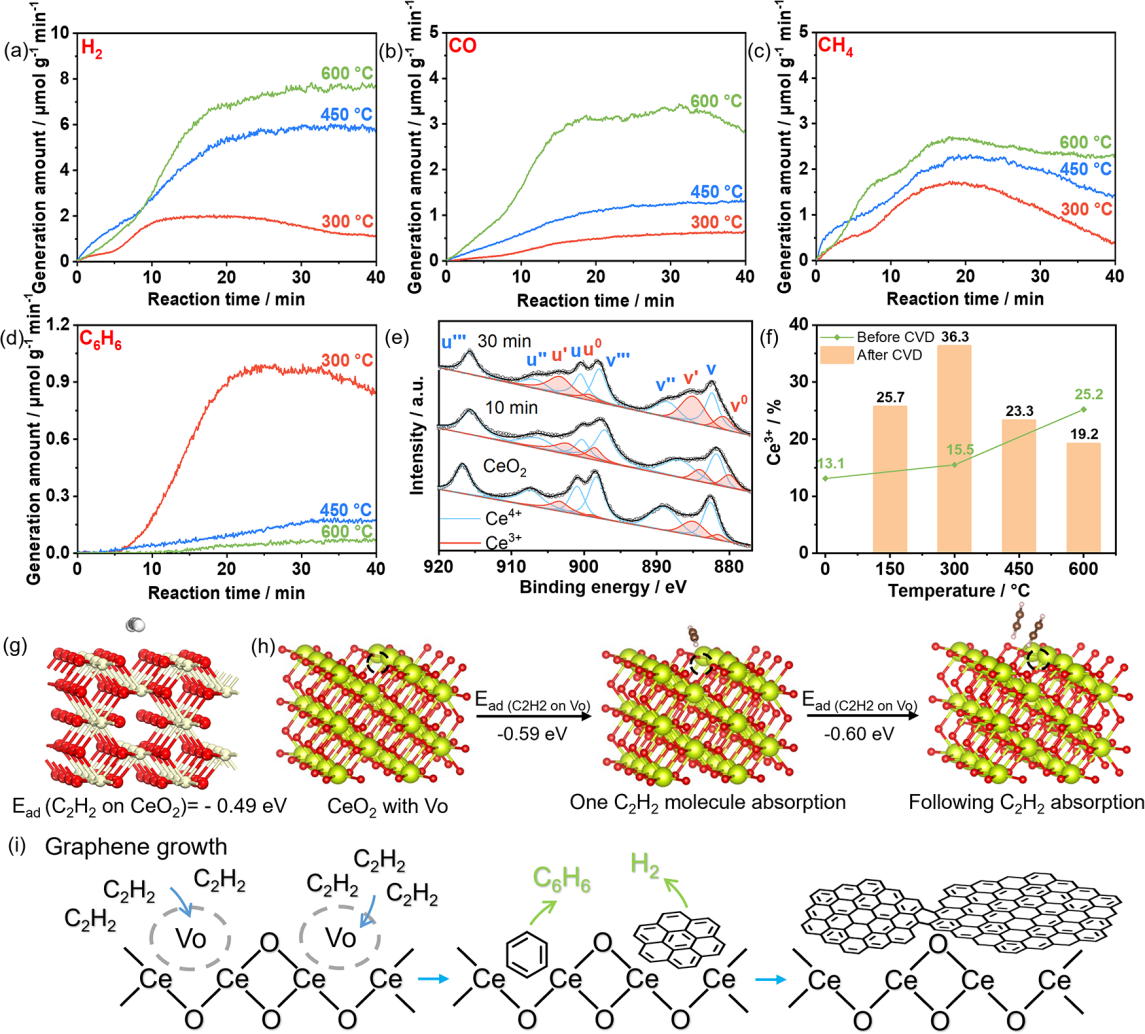
Gas emission of (a) H_2_, (b) CO, (c) CH_4_, and (d) C_6_H_6_ during C_2_H_2_ decomposition on CeO_2_ at different temperatures. C_2_H_2_ (1 vol % in He) was introduced to the reactor at 0 min. (e) Ce 3d XPS spectra for CeO_2_ before and after C_2_H_2_–CVD for 10 and 30 min at 300 °C. (f) Ce^3+^ ratio before and after C_2_H_2_–CVD calculated by Ce 3d XPS spectra. The adsorption of C_2_H_2_ on (g) CeO_2_ (110) surface and (h) CeO_2_ (110) with oxygen vacancy. (i) Schematic for the conclusive mechanism of the C_2_H_2_ decomposition reaction on CeO_2_.

Next, we further investigate the dynamic surface changes of CeO_2_ during the CVD process. As the release of surface oxygen is accompanied by the reduction of Ce^4+^ to Ce^3+^, the concentration of V_O_ can be correlated with the Ce^3+^ content, which is quantifiable via X-ray photoelectron spectroscopy (XPS).^46,47^ As shown in Figure 4e, the spectrum of CeO_2_ before reaction can be deconvoluted into ten subpeaks derived from Ce^4+^ and Ce^3+^.^46,48,49^ The Ce^3+^ ratio (*C*_Ce^3+^_ [%]) was calculated using the following equation

3 $$C_{{\mathrm{Ce}}^{3+}}=A\left({\mathrm{Ce}}^{3+}\right)/\left[A\left({\mathrm{Ce}}^{3+}\right)+A\left({\mathrm{Ce}}^{4+}\right)\right]\times 100$$

where *A*(Ce^3+^) and *A*(Ce^4+^) are the total peak areas corresponding to Ce^3+^ and Ce^4+^ components, respectively (Table S1). *C*_Ce^3+^_ on pristine CeO_2_ is 9.2%, suggesting the presence of V_O_ in the natural state. When CeO_2_ was exposed to C_2_H_2_ for varying durations at 300 °C, the concentration of Ce^3+^ increased significantly, reaching 20.3% after 10 min and further rising to 26.5% after 30 min. It indicates that the formation of graphene sheets on CeO_2_ at 300 °C is accompanied by the generation of V_O_. Moreover, XPS analysis was conducted on CeO_2_ after thermal treatment under Ar at the target temperature for 30 min, as well as after subsequent exposure to C_2_H_2_ for 30 min without air exposure. As summarized in Figure 4f, the concentration of Ce^3+^ gradually increased with heating up to 600 °C (Figure S13a). However, following CVD at elevated temperatures, the surface concentration of Ce^3+^ was lower than that observed at 300 °C (Figure S13b). This decrease may be attributed to the involvement of multiple reaction pathways during C_2_H_2_ decomposition at higher temperatures, which will be discussed in detail later. To further confirm the catalytic role of V_O_ by directly modulating the defect density, CeO_2_ was pretreated under Ar at 450 °C for 1 h and subsequently exposed to C_2_H_2_ at 150 °C. As shown in Figure S13c, the pretreated CeO_2_ exhibited a markedly greater initial weight increase than the nonpretreated sample. Since Ar pretreatment increases the surface defect density, as evidenced by the higher Ce^3+^ fraction in XPS (see Figure S13a and Table S1), this comparison suggests that an increased V_O_ concentration promotes C_2_H_2_ dissociation and carbon deposition on CeO_2_.

As discussed above, V_O_ are formed on the CeO_2_ surface during the CVD process, and these V_O_ sites are expected to serve as active centers for graphene growth. This is further supported by calculations of V_O_ formation energies for CeO_2_ and MgO in the absence and presence of C_2_H_2_ (Table S2). Whereas the V_O_ formation energy on MgO is essentially insensitive to C_2_H_2_ adsorption, C_2_H_2_ thermodynamically stabilizes vacancy formation on CeO_2_, lowering the formation energy by 0.15 eV. This suggests that V_O_ are readily accessible on CeO_2_ under CVD conditions, motivating an evaluation of C_2_H_2_ binding at V_O_ sites. Accordingly, we calculated the adsorption energy of C_2_H_2_ around V_O_ and compared it with that on the defect-free CeO_2_ surface. C_2_H_2_ adsorption on the pristine surface requires −0.49 eV (Figure 4g), whereas adsorption around V_O_ is energetically more favorable, requiring only −0.59 eV (Figure 4h). Subsequent adsorption also remains favorable at −0.60 eV, suggesting that each V_O_ site can effectively adsorb and stabilize several C_2_H_2_ molecules, catalyzing their decomposition and the nucleation of graphene. Thus, these findings support the notion that V_O_ plays a critical role in graphene growth. We also considered the possibility that CO_2_-like surface structures formed by CO reabsorption serve as potential active sites (see Figure S13d). However, C_2_H_2_ adsorption around these CO_2_-derived structures is energetically unfavorable, requiring 0.84 eV (Figure S13d), which indicates that V_O_, rather than CO_2_-derived species, are the primary active sites responsible for promoting graphene growth. Note that large amounts of CO_2_-derived structures are probably formed at elevated temperatures, which consequently reduces the density of active V_O_ sites, hindering C_2_H_2_ adsorption and leading to the shortened duration of stage 1 observed at 600 °C (see Figure 2b).

Returning to the CVD-MS discussion, we now consider the graphene growth mechanism in detail. As shown in Figure 4d, benzene (C_6_H_6_) was clearly detected at 300 °C. While our DFT results indicate that the initial activation of C_2_H_2_ on CeO_2_ is kinetically dominated by a radical-type dissociative pathway that generates V_O_ (Figure 3a,c), this radical mechanism involving C–C bond cleavage appears implausible for the formation of C_6_H_6_. In contrast, previous studies support the idea that a carbene-mediated pathway can more reasonably account for the trimerization of C_2_H_2_ to form C_6_H_6_.^42,44^ Since the carbene-related route (Figure 3a) is kinetically less favorable than the radical route but remains energetically feasible, it should become accessible at elevated temperatures. We therefore use CVD-MS fragments to differentiate the two pathways (Figure S14): the radical pathway is associated with C_3_H_*x*_ species (*m*/*z* = 39/41), whereas the carbene-related route preferentially yields C_4_H_*x*_ species (*m*/*z* = 51) as C–C bond dissociation is not involved in the carbene-related route. Although coupling of C_3_H_*x*_ could in principle produce C_6_H_6_, the *m*/*z* = 39/41 kinetics at 300 °C differ from the C_6_H_6_ signal, whereas *m*/*z* = 51 closely tracks C_6_H_6_ even after correcting for C_6_H_6_ fragmentation (Figure S14a), indicating that C_6_H_6_ formation proceeds predominantly via the carbene-related pathway. Note that the C_6_H_6_ signal decreases markedly at 450 °C and is barely detectable at 600 °C (Figure 4d). Consistently, *m*/*z* = 51 gradually decreases from 300 to 600 °C (Figure S14b, c), whereas *m*/*z* = 39/41 increases from 300 to 450 °C. These declines do not indicate suppression of the reaction itself; rather, it implies that the intermediates continue to react, forming extended sp^2^ carbon networks and ultimately leading to graphene growth. This interpretation is supported by the disappearance of characteristic DRIFTS peaks (see Figure S7e).

CH_4_ was also produced during C_2_H_2_ decomposition, with its yield increasing as the temperature rose (Figure 4c). Notably, the production trend of CH_4_ closely follows that of H_2_, suggesting that CH_4_ formation may proceed via the reaction between C_2_H_2_ and H_2_. One plausible radical-based route involves the formation of CH_2_* intermediates, which may then react with surface hydrogen to form CH_3_* (Δ*E* = 2.34 eV), followed by CH_4_ formation (Δ*E* = 1.61 eV) (Figure S15). However, this radical pathway is energetically unfavorable due to the relatively high activation barriers involved. In contrast, when a carbene-mediated pathway is considered, the formation of CH_4_ can be explained in a more energetically favorable manner. The migrated H* tends to saturate the CCH_2_* intermediate, leading to the formation of a partially hydrogenated structure that subsequently produces CH_4_ with a significantly lower energy barrier (Figure S16). Combined with the mechanistic analysis of C_6_H_6_ formation, this result further supports the involvement of a carbene-mediated pathway at high temperatures.

Based on experimental observation and DFT calculations, a plausible mechanism for the conversion of C_2_H_2_ into graphene is illustrated in Figure 4i. Initially, owing to the high oxygen mobility of CeO_2_, C_2_H_2_ undergoes decomposition on the CeO_2_ surface via a radical pathway, accompanied by the formation of V_O_ (see Figure 3). These V_O_ sites promote further adsorption of C_2_H_2_ and facilitate its subsequent decomposition. At elevated temperatures, reduced kinetic barriers render the carbene-related coupling channel competitive, whereas radical-type dissociation remains primarily responsible for C_2_H_2_ activation and V_O_ generation. The synergy of these processes promotes the growth of extended sp^2^ carbon frameworks and ultimately graphene.

### 3.3 Oxygen-Vacancy–Directed Carbon Structure Evolution

As described above, the decomposition behavior of C_2_H_2_ is highly temperature dependent. Therefore, we attempted to synthesize graphene-based materials with distinct structures and properties by tuning the temperature, following the procedure illustrated in Figure 1. At lower temperatures, the reduced V_O_ density provides spatially isolated active sites for C_2_H_2_ activation, leading to controlled decomposition kinetics and selective carbon nucleation. This moderation in reactivity suppresses excessive carbon coupling and promotes the formation of quantum-confined graphene domains. At 300 °C, it leads to the generation of a graphene quantum dot (GQD) suspension through catalytic C_2_H_2_ decomposition on CeO_2_ followed by sonication in ethanol.

The TEM image of C/CeO_2_-300 shows that the composite retains the overall morphology of CeO_2_ (Figure S17a,b). The corresponding SAED pattern is dominated by diffraction rings from CeO_2_ (Figure S17c), with no clearly distinguishable contribution from graphitic carbon. After dispersion in ethanol, the resulting suspension exhibits a yellow color under ambient light and strong blue fluorescence under 365 nm UV irradiation (inset, Figure 5a). In addition, the representative UV–vis absorption spectrum of the GQDs (Figure 5a) displays two characteristic peaks at ∼252 nm and ∼285 nm, assigned to the π–π* transition of aromatic sp^2^ C=C domains and the *n*–π* transition of defect-related sp^3^ regions, respectively. The broad absorption band below 600 nm is attributed to surface states of the GQDs. Upon excitation, the GQD suspension exhibits a broad photoluminescence (PL) emission spanning 300–600 nm (Figures 5a and S18).

**Figure 5:**
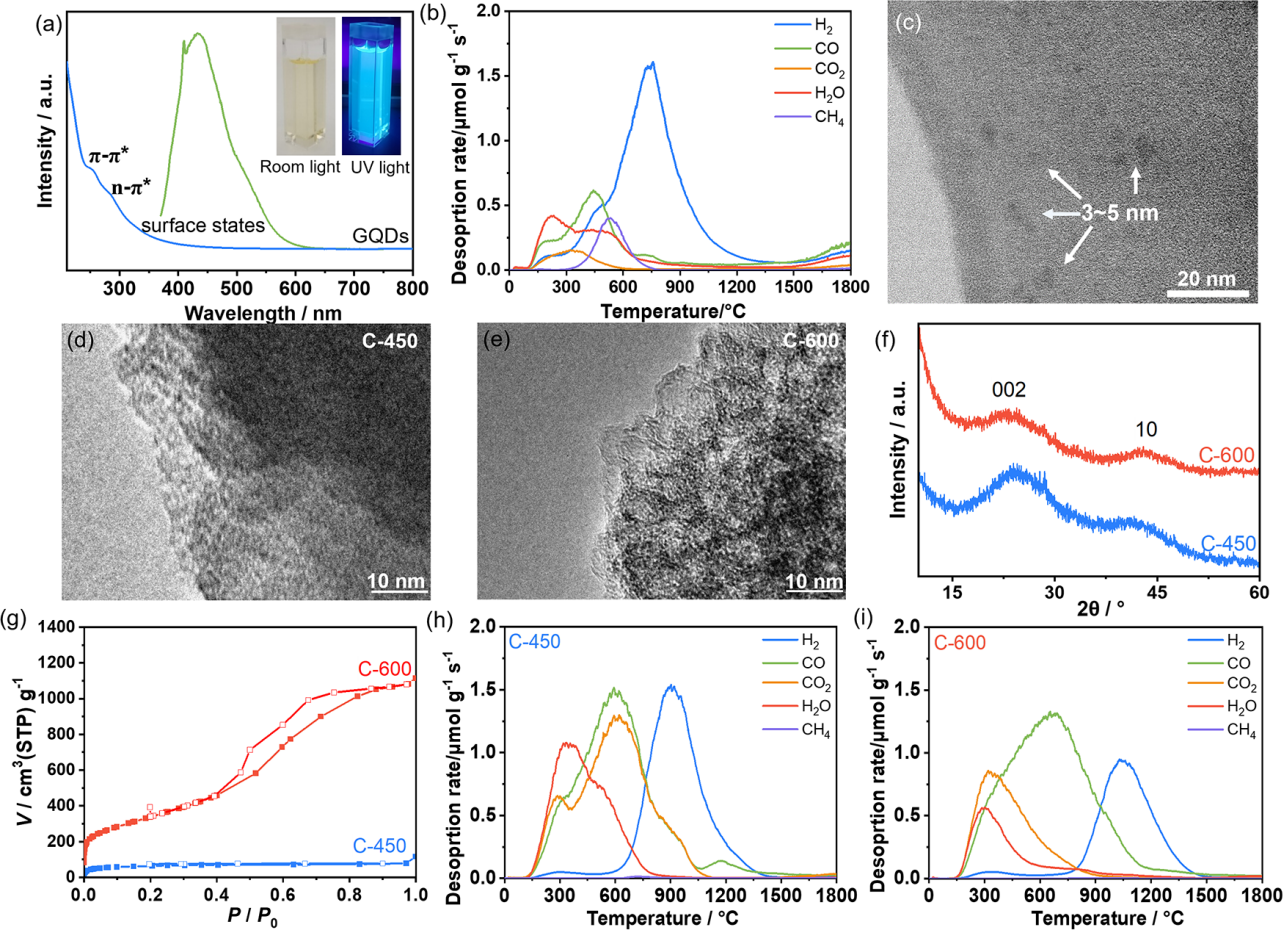
(a) UV–vis absorption spectra (blue line) and PL spectra (green line) excited at 365 nm of GQDs suspension. The inset in (a) is GQD suspensions under room light and UV light (365 nm). (b) TPD profiles and (c) bright field-STEM image of GQDs. TEM images of (d) C-450 and (e) C-600. (f) XRD patterns and (g) N_2_ adsorption/desorption profiles of C-450 and C-600. TPD profiles of (h) C-450 and (i) C-600.

The corresponding STEM image reveals the presence of GQDs with an average diameter of approximately 3 nm (Figures 5c and S19a–d). Compared with the blank carbon support film (Figure S19e), the sample-dispersed regions exhibit an enhanced C signal in the EDS analysis, while no Ce or other contamination related signals are detected (Figure S19f). Further structural insight is provided by HRTEM analysis. For the separated GQDs deposited on a carbon film, HRTEM images reveal locally ordered hexagonal (honeycomb) lattice structures characteristic of sp^2^ carbon (Figure S20a,b). The corresponding FFT patterns exhibit distinct hexagonal diffraction features. In contrast, the empty support film shows no such hexagonal features (Figure S20c). These results provide direct evidence for the formation of graphene-based sp^2^ carbon domains under mild conditions (300 °C).

High-sensitivity vacuum temperature-programmed desorption (TPD) up to 1800 °C was performed for GQDs, as shown in Figure 5b. During this process, hydrogen- and oxygen-terminated edge sites decomposed, releasing as H_2_, CO, CO_2_, H_2_O and CH_4_. Accordingly, the total number of edge sites (*N*_edge_) and the average domain size of graphene (*S*_G_) can be calculated using eqs 4 and 5,^50^ in which *a* [nm] is the sp^2^ C–C bond length:

4 $$N_{\mathrm{edge}}=2N_{H_{2}}+2N_{H_{2}O}\left(>400^{{}^{\circ}}C\right)+N_{\mathrm{CO}}+2N_{{\mathrm{CO}}_{2}}+2N_{{\mathrm{CH}}_{4}}$$

5 $$S_{G}=a/6N_{\mathrm{edge}}$$

The *N*_edge_ of GQDs was determined to be 11.0 mmol g^–1^, corresponding to an estimated *S*_G_ of 3.7 nm, which is in good agreement with the average diameter of 3–5 nm observed in the multiple bright-field STEM image (Figures 5c and S19a–d) and HRTEM image (Figure S20). Notably, the predominant desorbed species was H_2_, with only minor amounts of CO and CO_2_, indicating that the majority of edge sites are hydrogen-terminated. In addition, the formation of a small amount of CH_4_ suggests the presence of sp^3^-hybridized carbon terminations at the edges. Conventional top-down methods for preparing GQDs typically involve oxidative cutting of graphite or graphene using large quantities of strong acids or oxidants, which inevitably generate oxygen-rich edge functionalities that deteriorate the intrinsic properties of the resulting materials.^51,52^ In contrast, the present approach affords GQDs with a low concentration of oxygen-terminated edges under mild and environmentally benign conditions.

For C/CeO_2_-450 and C/CeO_2_-600, the CeO_2_ template was removed using a mixture of HNO_3_ and H_2_O_2_ to obtain the corresponding carbon materials, denoted as C-450 and C-600, respectively. TEM analysis reveals that C-450 (Figure 5d) exhibits a stacked morphology significantly differing from C/CeO_2_-450 prior to template removal (Figure S21), indicating the collapse of template-derived structure at this temperature. In contrast, C-600 (Figures 5e and S22a) displays a continuous and ordered porous structure, closely resembling that of C/CeO_2_-600. This suggests that the structural features from the template are preserved when the carbonization temperature is increased to 600 °C by enhancing carbon connectivity. The corresponding SAED pattern exhibits distinct diffraction rings with *d*-spacings of ∼0.21 and ∼0.12 nm, assignable to the (10) and (11) planes of graphitic carbon, respectively (Figure S22b). Both samples exhibit similar XRD patterns characterized by two distinct peaks characteristic of turbostratic sp^2^ carbon (Figure 5f). The weak and broad peak around 2θ = 24°, indexed to the 002 reflection, corresponds to the stacking of graphene sheets, while the peak at 2θ = 43°, assigned to the 10 planes, arises from in-plane diffraction of graphene layers. The full width at half-maximum (FWHM) of the two peaks were used to assess stacking and in-plane coherence. Compared with C-450, C-600 displays a broader (002) but a narrower (10) reflection, indicating larger laterally coherent sp^2^ domains at 600 °C accompanied by a reduced degree of interlayer stacking.^10,36^ Raman spectra further support this tendency (Figure S23). Both samples display the characteristic D (∼1350 cm^–1^) and G (∼1580 cm^–1^) bands, whereas the 2D band is not clearly resolved, consistent with a stacking-disordered (turbostratic) framework. Notably, C-600 shows sharper D and G bands, suggesting improved local graphitic ordering. The increase in the *I*_D_/*I*_G_ ratio from C-450 to C-600 is attributed to the enhanced interconnection between domains.^53^ These collectively suggest that C-600 develops a more interconnected and laterally coherent sp^2^ carbon framework, enabling retention of the porous architecture upon template removal (Figure 5e), whereas the stacked carbon formed at 450 °C is susceptible to collapse (Figure 5d). To clarify the structural nature of the carbon products formed at different temperatures, C 1s XPS was further performed on the carbon-coated CeO_2_ composites obtained at 300, 450, and 600 °C (Figure S24). In all three samples, the C=C component at ∼284.4 eV is dominant, whereas the sp^3^ fraction remains limited (∼15%), indicating that the carbon products remain sp^2^-dominated across the entire temperature range.

The N_2_ adsorption isotherm further highlights the influence of catalytic dynamics on graphene framework formation. C-450 reveals a relatively low specific surface area of 178 m^2^ g^–1^ and exhibits no apparent porosity (Figure 5g), whereas C-600 displays a high specific surface area of 1448  m^2^ g^–1^ and a distinct hysteresis loop at *P*/*P*_0_ > 0.3, indicative of mesoporosity and enhanced connectivity. These results suggest that the structural disruption during the removal process in C-450 is presumably caused by its insufficient structural stability, which originates from differences in temperature-dependent growth behavior, leading to variations in structural morphology.

Furthermore, *N*_edge_ and *S*_G_ of C-450 and C-600 were also estimated by TPD, as shown in Figure 5h,i. The *N*_edge_ values of C-450 and C-600 were determined to be 14.9 and 11.3 mmol g^–1^, respectively, corresponding to calculated domain sizes of 2.7 and 3.7 nm. Note that *S*_G_ describes the average size of individual sp^2^ graphene sheets. These sheets are laterally interconnected to form a larger turbostratic carbon framework whose overall dimensions, as observed by TEM, are much larger. Compared with GQDs, both C-450 and C-600 exhibited higher CO and CO_2_ evolution between 300 and 1200 °C, suggesting the higher concentration of terminal oxygen functionalities introduced during template removal with HNO_3_/H_2_O_2_. Notably, although the domain sizes of GQDs, C-450, and C-600 were not significantly different, their morphologies differed markedly. This clearly suggests that morphology cannot be evaluated solely based on domain size; rather, the interconnection between domains must also be considered. Therefore, tuning the CVD temperature from 300 to 600 °C enables control of the extent of graphene structure growth, or more precisely, the degree of interconnection between graphene domains. This temperature-dependent modulation of domain connectivity plays a crucial role in shaping the final structure after template removal, giving rise to a diverse range of morphologies, from discrete graphene (GQDs) to fully interconnected 3D porous graphene frameworks that replicate the original template structure. This arises from the synergistic coupling between carbon precursor activation and the redox dynamics of the oxide template. Such interplay enables acetylene decomposition and carbon growth to proceed at remarkably low temperatures (∼300 °C), establishing a defect-mediated design principle for controlling sp^2^ carbon dimensionality and framework morphology.

To correlate the temperature-dependent framework connectivity with charge transport, the electrical conductivity of the as-grown composites (C/CeO_2_-300, C/CeO_2_-450, and C/CeO_2_-600) was measured prior to template removal to avoid any etching-related effects. Conductivity was monitored using a two-probe method under uniaxial compression (0–50 MPa; Figure S25). Across the entire pressure range, C/CeO_2_-600 exhibits substantially higher conductivity than C/CeO_2_-450. At 50 MPa, the conductivity of C/CeO_2_-600 reaches 3.2 S cm^–1^, approximately 3 orders of magnitude higher than that of C/CeO_2_-450 (∼2.7 × 10^–3^ S cm^–1^). The markedly higher conductivity observed for C/CeO_2_-600 further supports the formation of an electrically percolated sp^2^ carbon network at 600 °C. This interpretation is consistent with the enhanced structural integrity and the retention of the porous architecture observed after template removal. By comparison, the conductivity of C/CeO_2_-300 remained below the detection limit of our setup, suggesting that while the carbon formed at 450 °C possesses a certain degree of electrical connectivity even without reaching 600 °C, the carbon generated at 300 °C exists in a spatially discrete manner.

CeO_2_ is a structurally tunable oxide whose morphology can be deliberately engineered to control the exposed crystal facets. This feature not only enables further corroboration of the structure sensitivity of the vacancy-mediated catalysis, but also offers an opportunity for morphology-controlled carbon synthesis. Accordingly, CeO_2_ with defined morphologies was employed as facet-selective templates. CeO_2_ nanorods and nano-octahedra (denoted as CeO_2_-rod and CeO_2_-octa) were synthesized via a hydrothermal route, predominantly exposing the (110) and (111) facets, respectively (Figure S26).^54,55^ The onset temperature for C_2_H_2_-to-carbon conversion is 117 °C on CeO_2_-rod and increases to 133 °C on CeO_2_-octa (Figure S27), indicating higher activity on the (110) facet than on the (111) facet. This trend aligns with the well-established higher reducibility of CeO_2_ (110) and its lower barrier to V_O_ formation relative to CeO_2_ (111), identifying V_O_ generation as a key determinant for carbon nucleation. Furthermore, C_2_H_2_–CVD at 600 °C shows a higher carbon growth rate on CeO_2_-rod than on CeO_2_-octa (Figure S28a), suggesting that (110)-dominated surfaces more effectively promote C_2_H_2_ decomposition and carbon growth. Overall, the morphology-controlled results establish that both the initiation and growth of carbon on CeO_2_ are strongly facet-dependent, with V_O_ formation propensity as a key contributing factor. For representative verification of the template-replica carbon, the CeO_2_-rod sample after CVD at 600 °C was etched to remove the CeO_2_ template, yielding a rod-templated tubular carbon (denoted as C-rod) (Figure S28b).

From a practical perspective, this CeO_2_-based CVD process is compatible with scale-up using established CVD reactors. As a proof of concept, a 10 g-scale run in a desktop rotary kiln furnace was performed at 600 °C to yield carbon-coated CeO_2_ (Figure S29a). After etching, porous carbons with a comparable characteristic morphology were obtained (Figure S29b), suggesting potential for larger-batch operation and continuous production with appropriate engineering design.

## 4 Conclusions

A series of graphene-based materials with distinct structural and physicochemical properties were successfully synthesized by modulating the growth conditions during catalytic C_2_H_2_ decomposition over CeO_2_. Combination of *in situ* characterization techniques and density functional theory calculations revealed that C_2_H_2_ activation proceeds through a Vo–mediated catalytic process, dynamically coupling hydrocarbon activation with lattice oxygen migration. This defect-mediated pathway enables C_2_H_2_ to decompose on the CeO_2_ surface at an exceptionally low temperature of 113 °C, while sp^2^ carbon structure formation occurs below 300 °C through a two-stage C_2_H_2_ chemical vapor deposition process. During the initial activation step, C_2_H_2_ interacts with the CeO_2_ surface and undergoes dissociative adsorption and reorganization, forming surface-bound HCHO and CO. The desorption of these species generates oxygen vacancies, which serve as active sites to promote the nucleation and growth of graphene sheets at low temperatures. The tunability of Vo density with temperature governs the connectivity of sp^2^ domains, producing graphene quantum dots at 300 °C, aggregated graphene at 450 °C, and porous graphene frameworks at 600 °C. This temperature-dependent evolution reflects a designable transition in carbon dimensionality, governed by defect chemistry that links structural architecture with electronic functionality. Overall, this study establishes a defect-engineering paradigm in which oxygen-vacancy dynamics govern carbon growth and structural evolution, providing a general and sustainable strategy for the low-temperature synthesis of graphene-based nanomaterials on reducible oxides.

## Supplementary Material





## Notes

XRD pattern of CeO_2_ nanoparticles; TEM images for prepared samples; *in situ* CVD-TG profiles on different oxides; Arrhenius plots for *E*_a_ calculation; *in situ* CVD-MS, DRIFTS, Raman results on CeO_2_; Raman spectra, TG profiles for prepared samples; structural model, energy profile of DFT calculation; Ce 3d and C 1s XPS spectra of CeO_2_ and prepared samples; PL spectra, STEM images, EDS profiles, HRTEM images of GQDs; Electrical conductivity test results; Photograph of desktop rotary kiln furnace and prepared samples (PDF)
DFT-based AIMD simulations (Video S1) (MP4)

## Abbreviations

CVD: chemical vapor deposition
CeO_2_: ceria
C_2_H_2_: acetylene
CH_4_: methane
V_O_: oxygen vacancy
GQDs: graphene quantum dots
TG: thermogravimetry
DRIFTS: diffuse reflectance infrared Fourier transform spectroscopy
XPS: X-ray photoelectron spectroscopy
Raman: Raman spectroscopy
DFT: density functional theory
AIMD: ab initio molecular dynamics
VASP: Vienna ab initio simulation package
GGA: generalized gradient approximation
PBE: Perdew–Burke–Ernzerhof functional
TPD: temperature-programmed desorption
STEM: scanning transmission electron microscopy
TEM: transmission electron microscopy
HRTEM: high-resolution transmission electron microscopy
UV–vis: ultraviolet–visible spectroscopy
PL: photoluminescence
*E*_a_: activation energy
*E*_ads_: adsorption energy
CMS: carbon mesosponge
GMS: graphene mesosponge
SSA: specific surface area
BET: Brunauer–Emmett–Teller
Ar: argon
He: helium
