# Supplementary material for: Defect-Mediated Catalysis for Low-Temperature Formation of Graphene-Based Materials
Source: J Am Chem Soc. 2026 Jun 8;148(25):25389–401. doi: 10.1021/jacs.5c20150 (PMC13339635; doi:10.1021/jacs.5c20150)
Supplement: Supplementary file 1 [file ja5c20150_si_001.pdf]

Supporting Information

Defect-Mediated Catalysis for Low-Temperature Formation of Graphene-based Materials

Mengxuan Zhang,† Takeharu Yoshii,, † Qi Zhao,‡ Yuichiro Hayasaka,§ Devis Di Tommaso,‡*

Hirotomo Nishihara,, †,#*

[†]Institute of Multidisciplinary Research for Advanced Materials, Tohoku University, 2-1-1 Katahira, Aoba-ku, Sendai, Miyagi, 980-8577, Japan.

[‡]School of Physical and Chemical Sciences, Queen Mary University of London, Mile End Road, London E1 4NS, UK

[§]The Electron Microscopy Centre, Tohoku University, 2-1-1 Katahira, Aoba, Sendai, Miyagi 980-8577, Japan

[#]Advanced Institute for Materials Research (WPI-AIMR), Tohoku University, 2-1-1 Katahira, Aoba-ku, Sendai, Miyagi, 980-8577, Japan.

*Email: takeharu.yoshii.b3@tohoku.ac.jp; hirotomo.nishihara.b1@tohoku.ac.jp

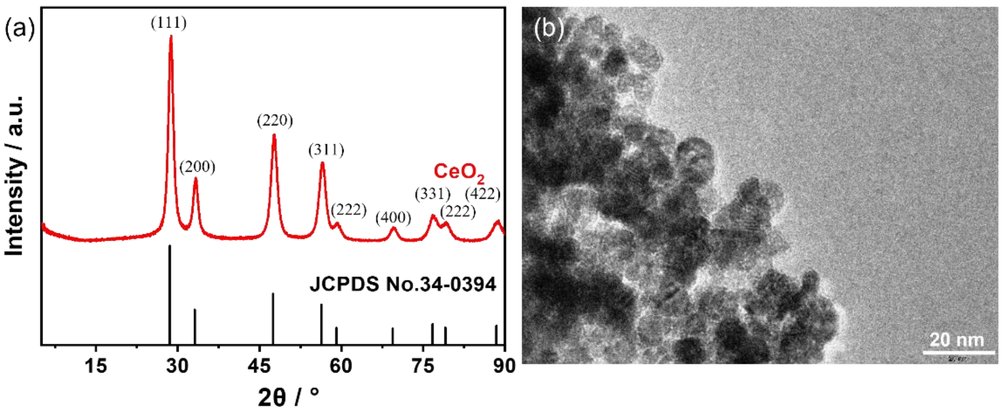

Figure S1. (a) XRD pattern and (b) TEM image of CeO_2. The characteristic diffraction peaks in (a) located at 28.5°, 33.1°, 47.5°, 56.4°, 59.1°, 69.4°, 76.1°, 79.1°, and 88.4° correspond to the lattice planes of (111), (200), (220), (311), (222), (400), (331), (420) and (422) of the typical fluorite structure of CeO_2, respectively.

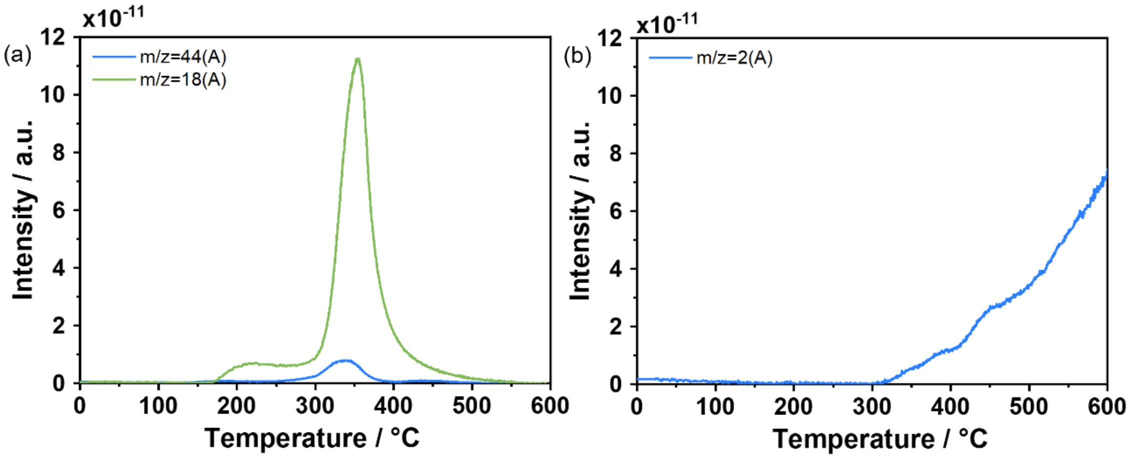

Figure S2. (a) MS ion-current profiles (baseline-subtracted raw intensities) of m/z = 44, 18 and (b) m/z = 2, recorded during temperature ramping on MgO under a mixture gas flow of C_2H_2 (1 v% in He).

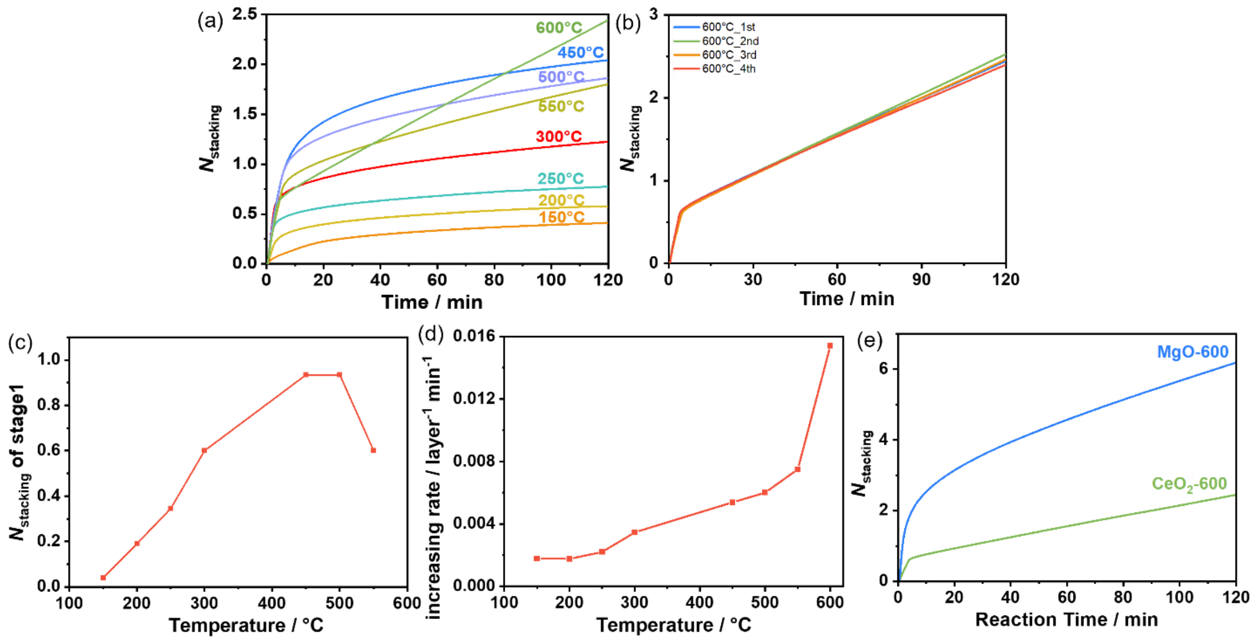

Figure S3. (a) Weight changes during catalytic C_2H_2 decomposition on CeO_2 at different temperatures and (b) reproducibility of weight change of CeO_2 at 600 ℃ monitored by TG. C_2H_2 was introduced to the reactor at 0 min. (c) $N_{stacking}$ in stage 1 under different temperatures. (d) Growth rate in stage 2 under different temperatures. (e) Weight changes of CeO_2 and MgO during C_2H_2-CVD at 600 ℃ monitored by TG. C_2H_2 was introduced to the reactor at 0 min. A mixture gas flow of C_2H_2 (20 v%) and Ar was used in all conditions.

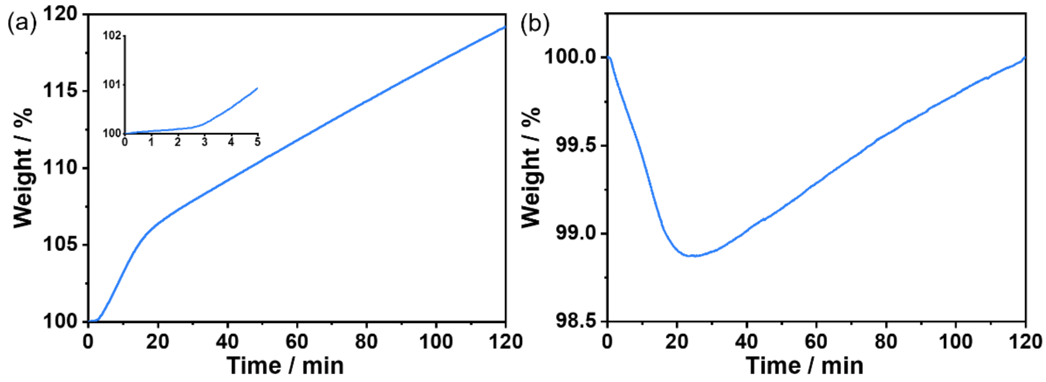

Figure S4. Weight change of (a) MgO and (b) CeO_2 under a mixture gas flow of CH_4 (20 v%) and Ar monitored by TG at 900 °C. The inset in (a) shows an enlarged view of the initial stage.

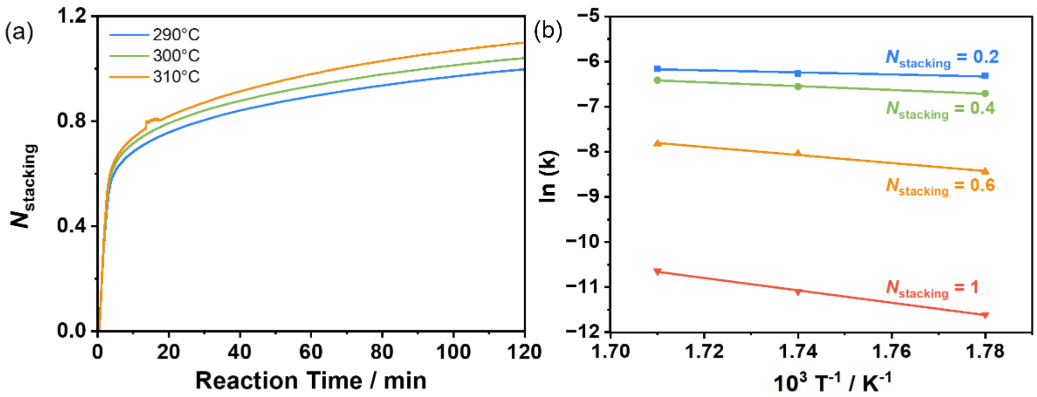

Figure S5. (a) Weight changes of CeO_2 at different temperatures under a mixture gas flow of C_2H_2 (20 v%) and Ar monitored by TG. (b) Arrhenius plots of catalytic C_2H_2 decomposition on CeO_2 at 300 °C in different periods of time that correspond to different $N_{stacking}$, where k is reaction rate (min^{-1}). The lines in (b) demonstrate a trendline fitting with a linear equation.

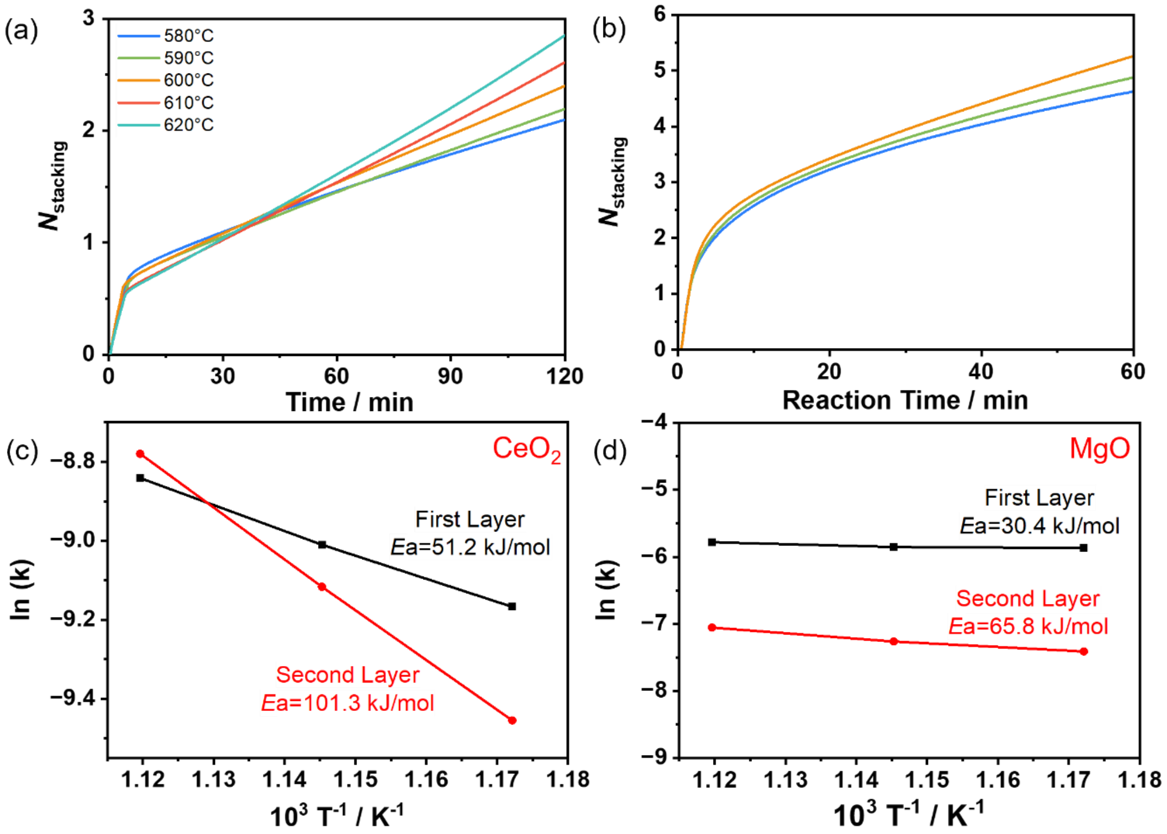

Figure S6. Weight changes of (a) CeO_2 and (b) MgO at different temperatures under a mixture gas flow of C_2H_2 (20 v%) and Ar monitored by TG. Arrhenius plots for the first- and second-layer deposition on (c) CeO_2 and (d) MgO at 600 °C, where k is reaction rate (min^{-1}). The lines in (c) and (d) demonstrate a trendline fitting with a linear equation.

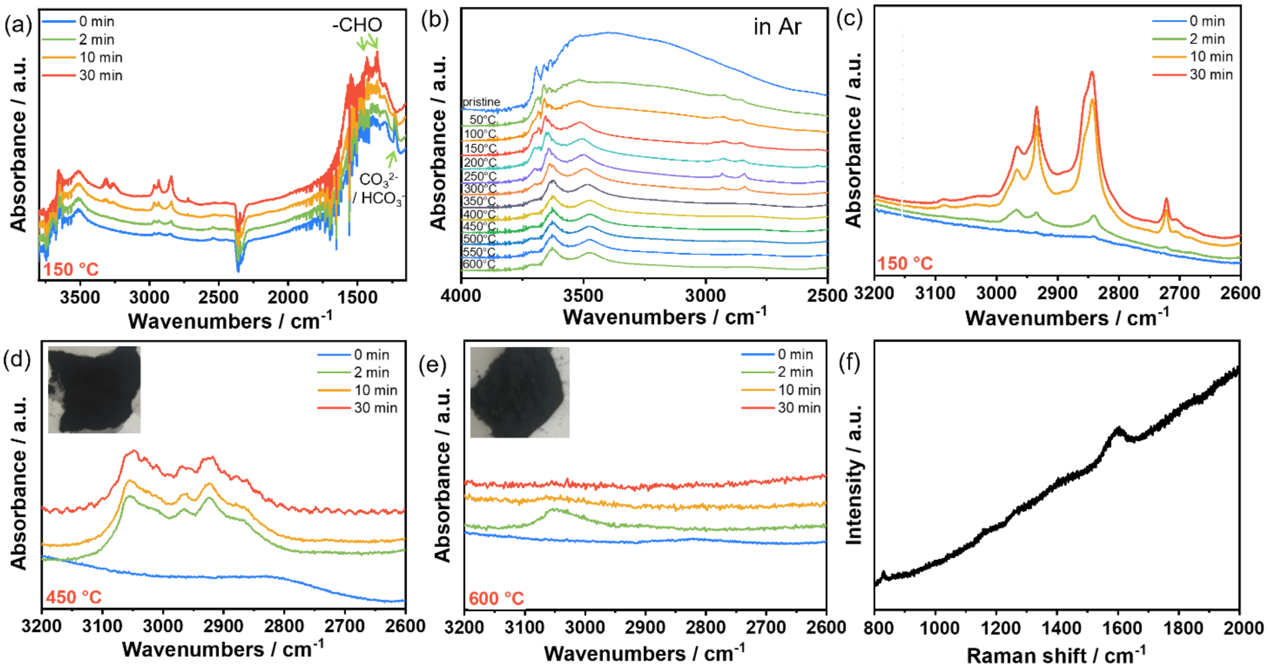

Figure S7. (a) *In situ* DRIFTS during catalytic C_2H_2 decomposition on CeO_2 at 150 °C. (b) *In situ* DRIFTS on CeO_2 under increasing temperature with the introduction of Ar. (c) *In situ* DRIFTS on CeO_2 under 150 °C with the introduction of C_2H_2 (1 v% in Ar) after pretreatment at 600 °C in Ar. *In situ* DRIFTS on CeO_2 for C_2H_2-CVD under (d) 450 °C and (e) 600 °C. The insets in (d) and (e) are sample images of C/CeO_2 after DRIFTS. (f) Raman spectrum before background subtraction of C/CeO_2 after DRIFTS at 300 °C.

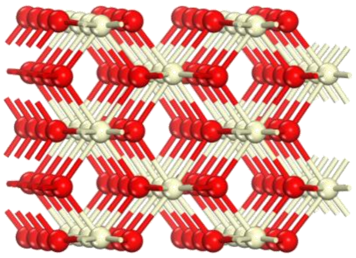

Figure S8. Structural model of the CeO_2 (110) surface.

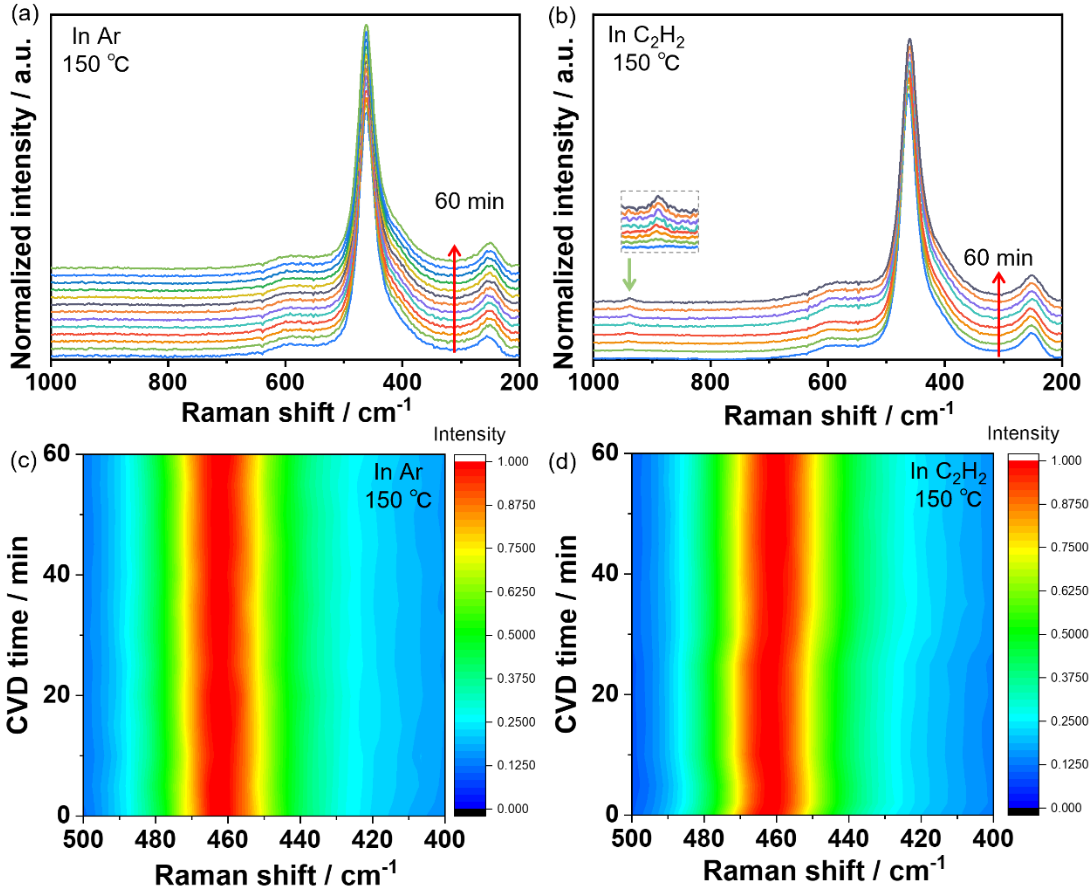

Figure S9. *In situ* Raman on CeO_2 at 150 °C with the introduction of (a) Ar and (b) mixture of C_2H_2 (1 v% in Ar). The inset in (b) shows an enlarged view of the band at around 937 cm^{-1}. (c, d) Corresponding time-wavenumber 2D intensity maps derived from (a) and (b), respectively, showing the evolution of the F_{2g} band region.

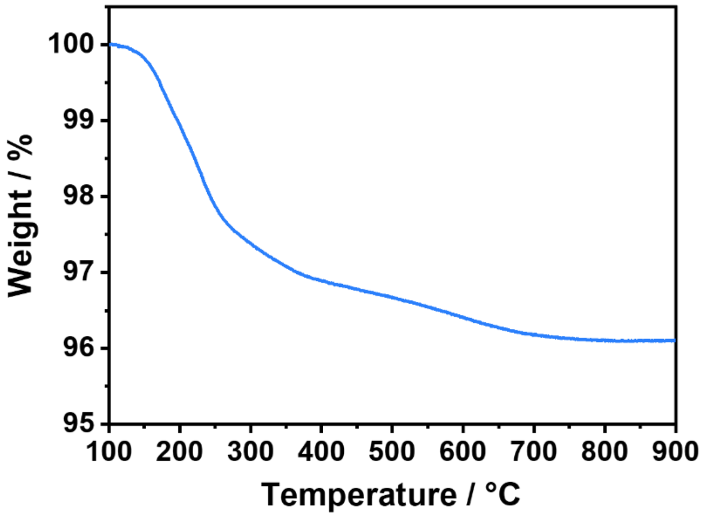

Figure S10. TG profile measured under air for C/CeO_2 after CVD at 150 °C.

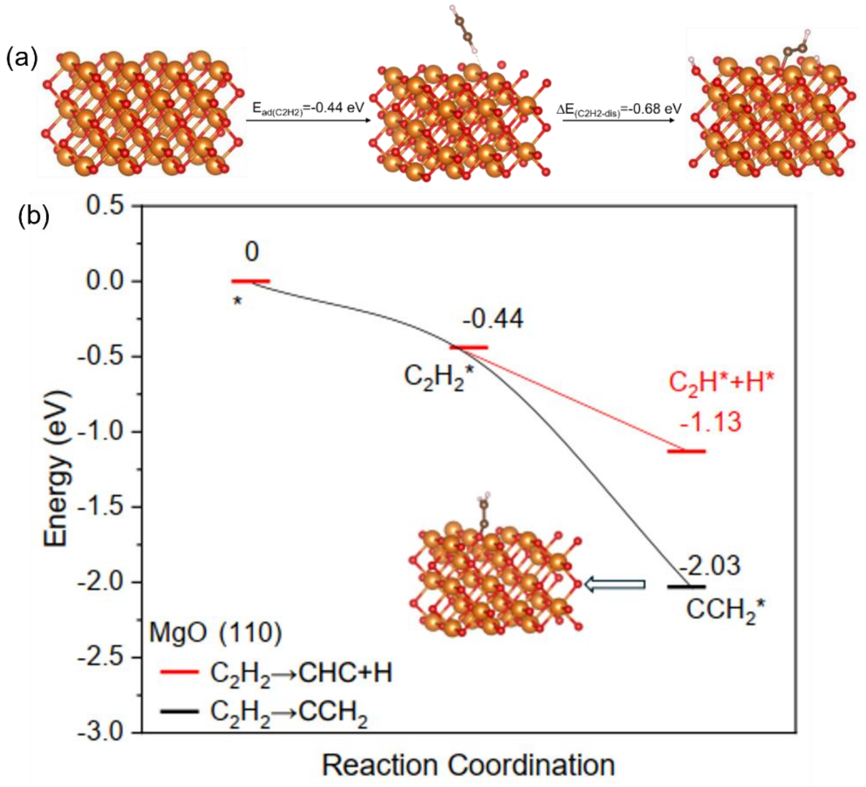

Figure S11. (a) The adsorption of C_2H_2 on MgO. (b) The energy profile of the C_2H_2 conversion on MgO (110) surface.

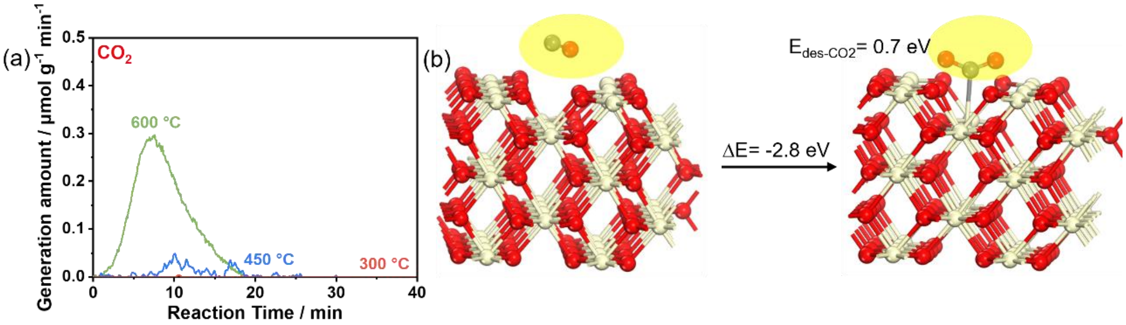

Figure S12. (a) CO_2 gas emission during catalytic C_2H_2 decomposition on CeO_2 at different temperatures. C_2H_2 (1 v% in He) was introduced to the reactor at 0 min. (b) CO-to-CO_2 conversion and energy changes on CeO_2 surface.

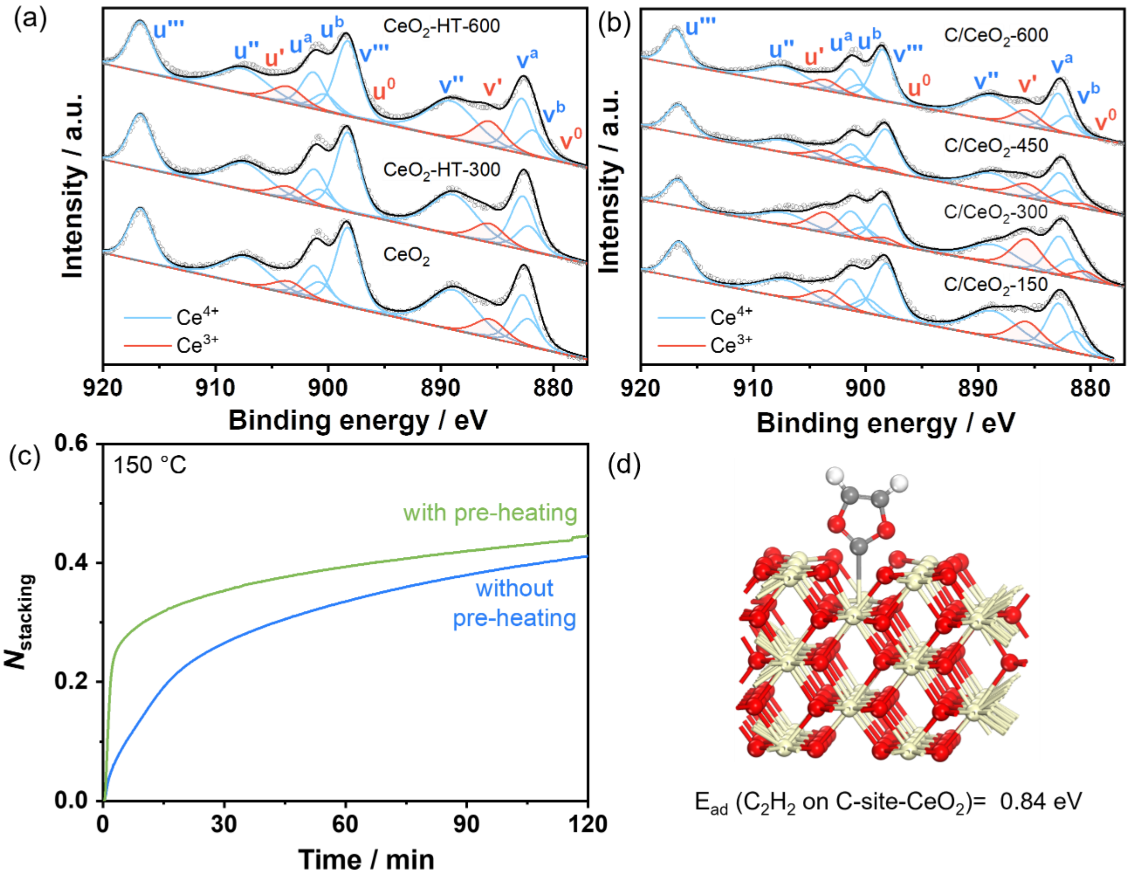

Figure S13. Ce 3d XPS spectra of CeO_2 (a) after heat treatment in Ar at different temperatures and (b) after C_2H_2-CVD at different temperatures. (c) Weight changes during catalytic C_2H_2 decomposition at 150 ℃ on CeO_2 with and without pre-heating at 450 ℃. (d) The adsorption energy of C_2H_2 on C-site of CeO_2.

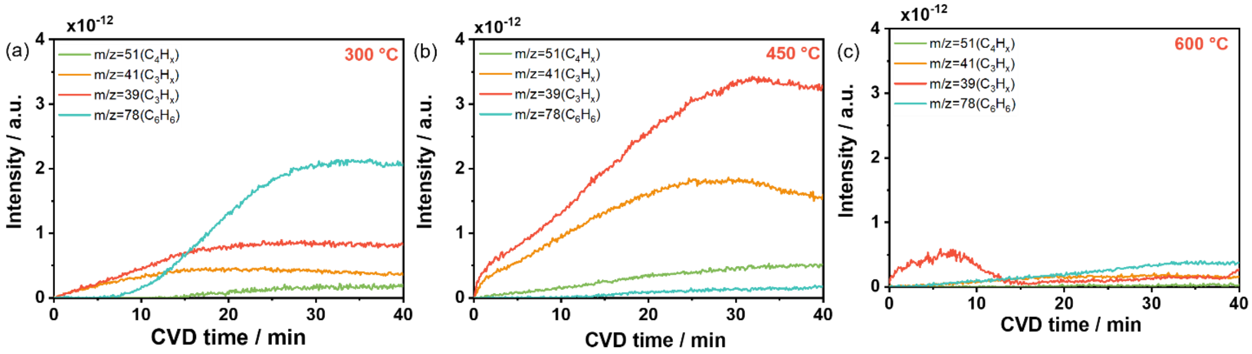

Figure S14. MS ion-current profiles (baseline-subtracted raw intensities) of m/z = 39, 41, 51, and 78, recorded during C_2H_2 decomposition on CeO_2 at (a) 300 °C, (b) 450 °C and (c) 600 °C. C_2H_2 (1 v% in He) was introduced to the reactor at 0 min.

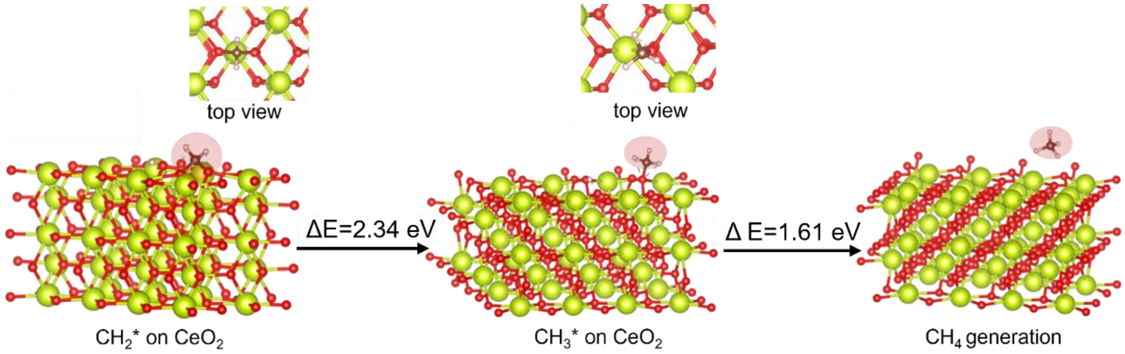

Figure S15. CH_4 formation on CeO_2 via the radical pathway.

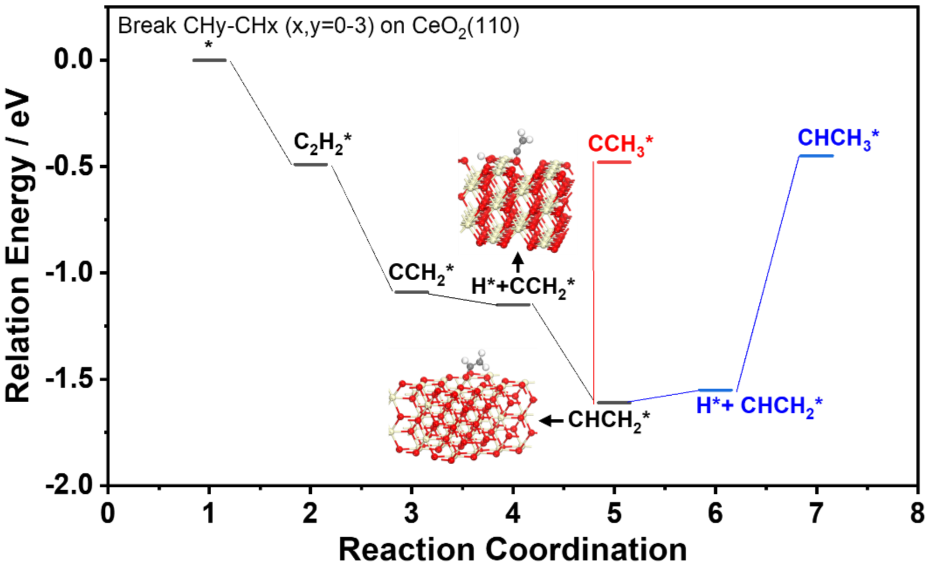

Figure S16. The energy profiles of the breakage of CH_y-CH_x (x, y = 0-3) on CeO_2 (110). The energy of CeO_2 surface before adsorption is set as 0 eV. The asterisk (*) denotes a molecule adsorbed on the CeO_2 surface.

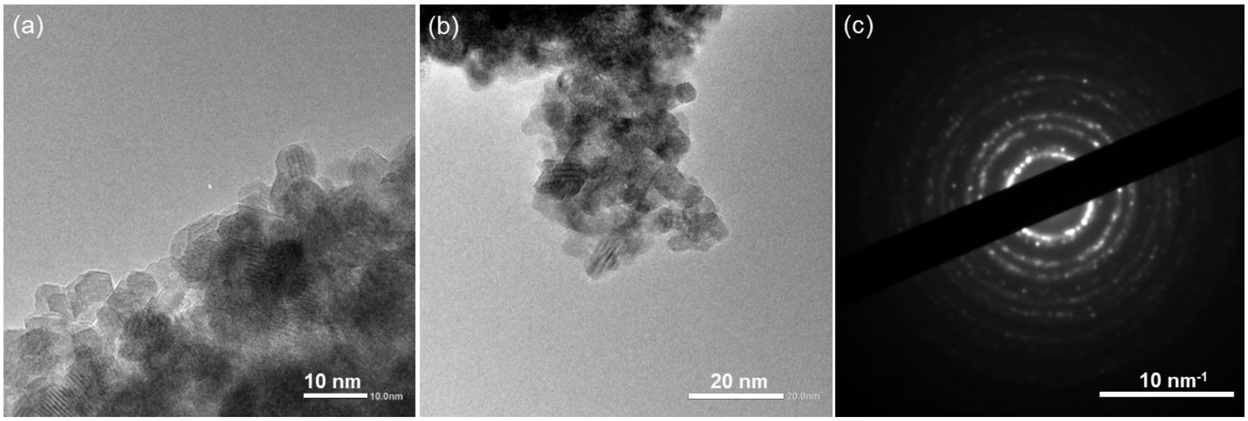

Figure S17. (a, b) TEM images and (c) the corresponding SAED pattern of C/CeO_2-300 composite.

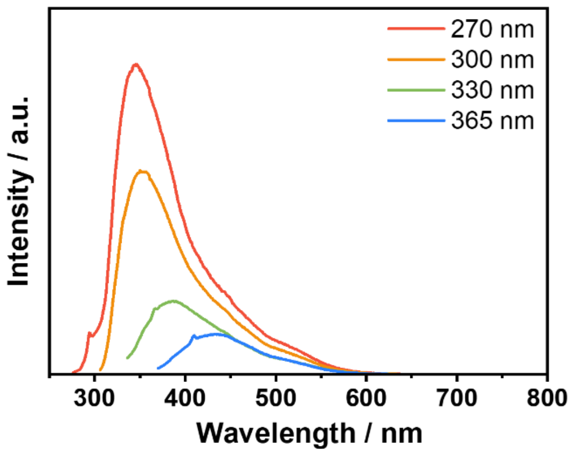

Figure S18. Photoluminescence spectra of GQD suspension under different excitation wavelengths.

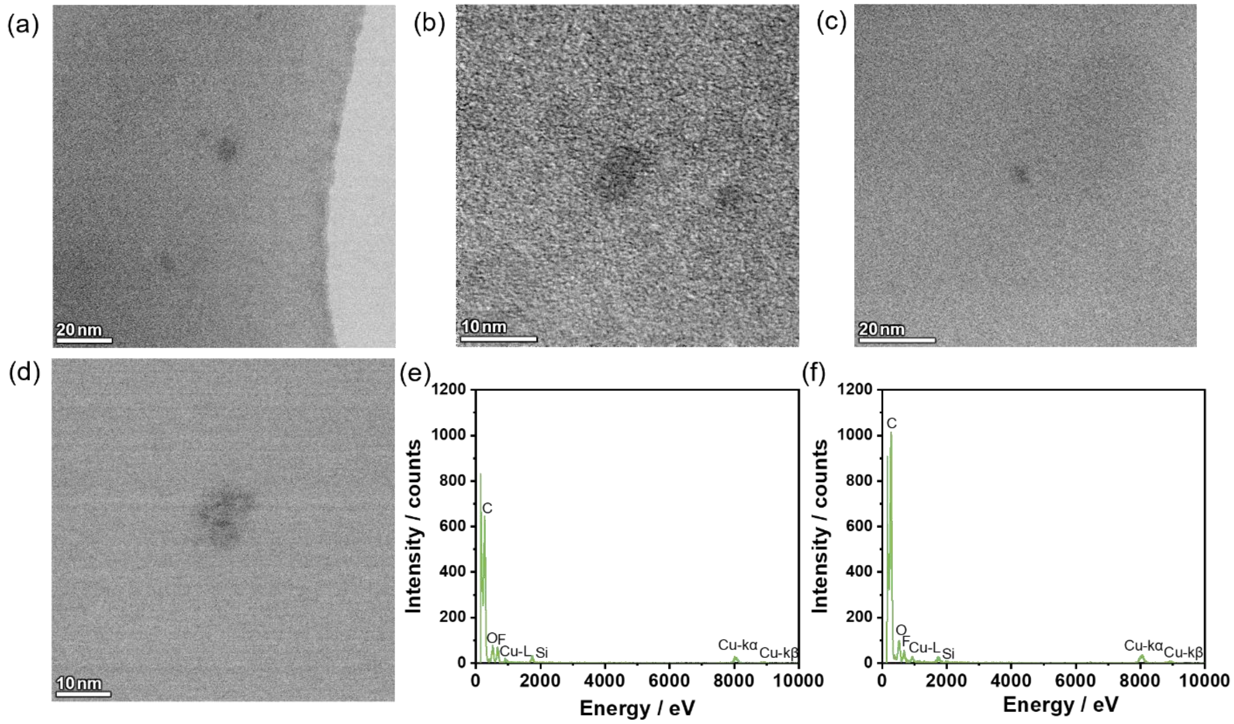

Figure S19. (a-d) Representative BF-STEM images of the GQD sample collected from different regions. EDS area averaged elemental spectra acquired from selected regions of (e) the blank support film and (f) the support film containing dispersed GQDs.

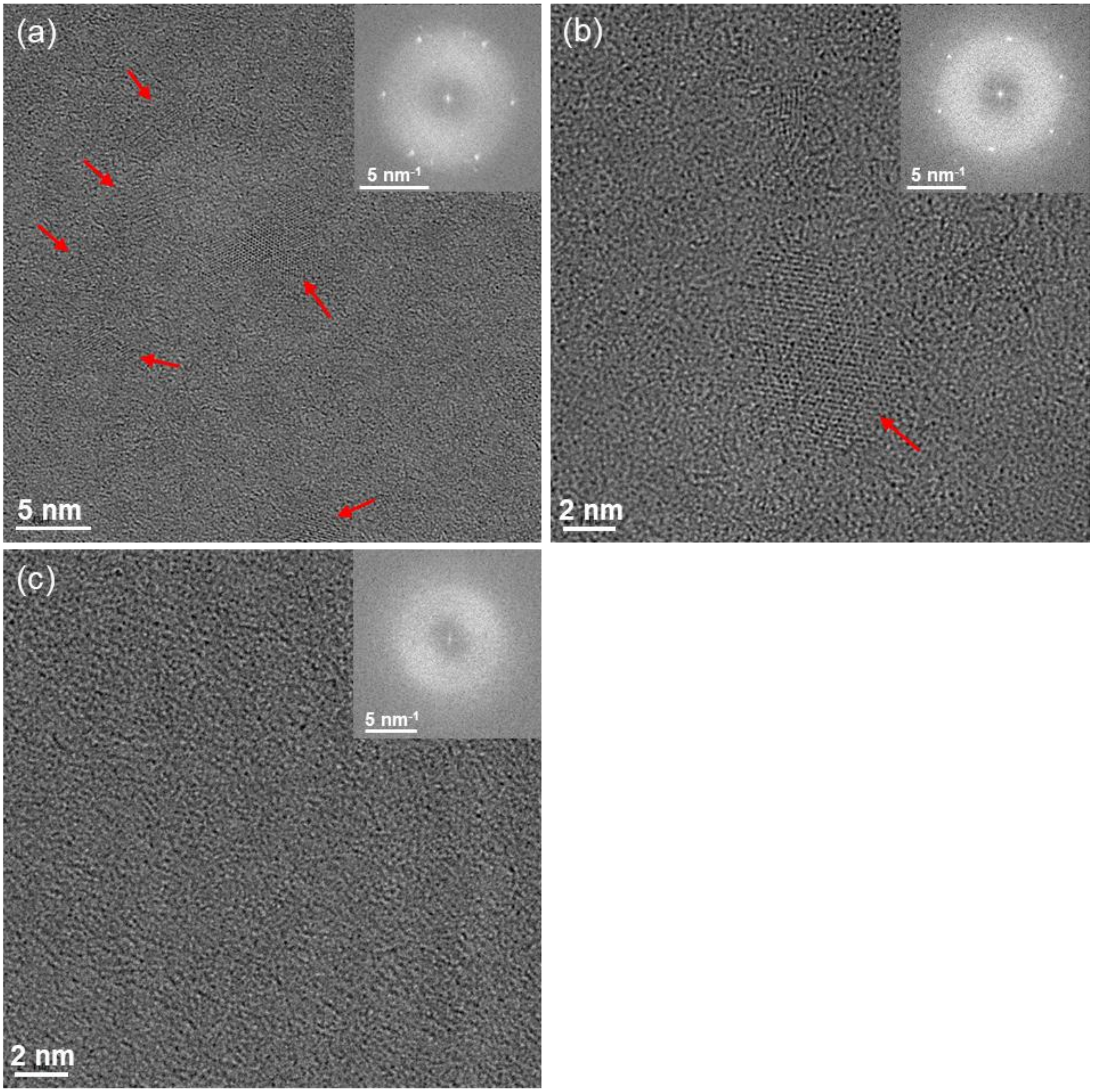

Figure S20. HRTEM images of (a, b) GQDs obtained from C/CeO_2-300 on a support film and (c) the empty support film. The insets in (a–c) show the corresponding FFT patterns.

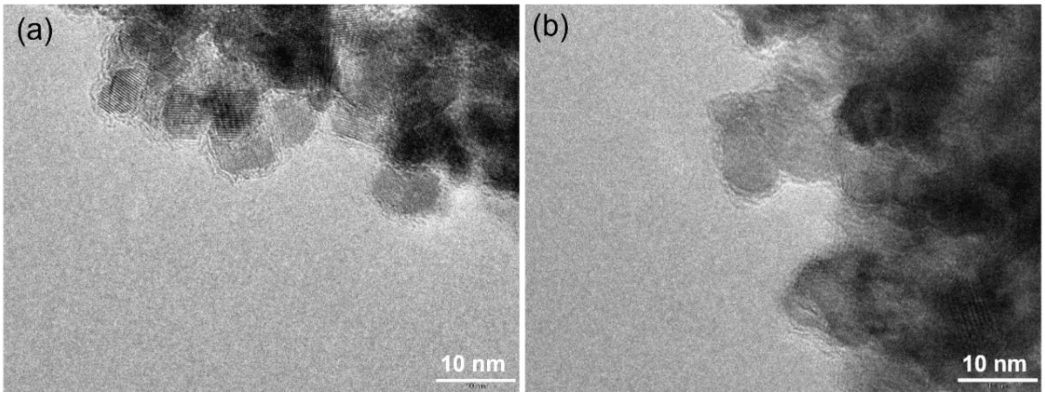

Figure S21. TEM images of (a) C/CeO_2-450 and (b) C/CeO_2-600.

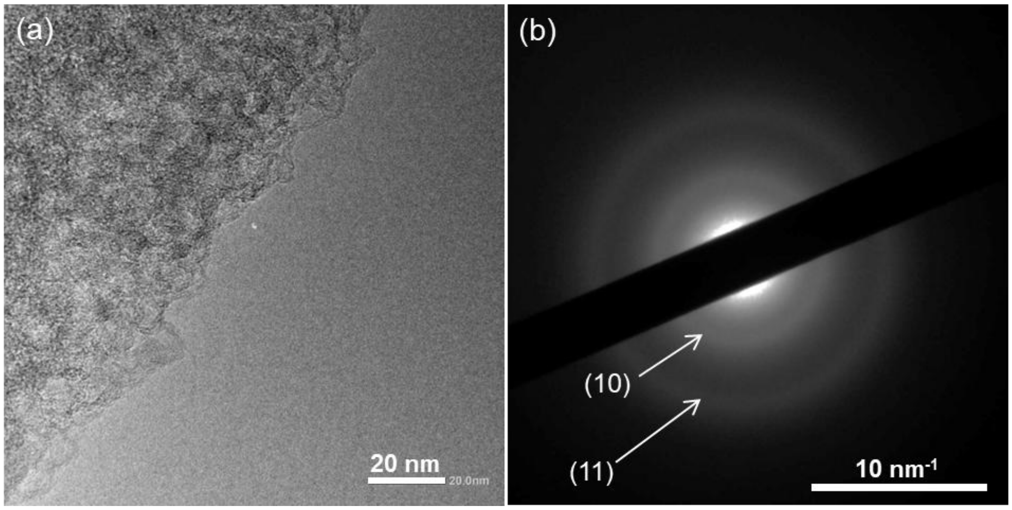

Figure S22. (a) TEM image and (b) the corresponding SAED pattern of C-600.

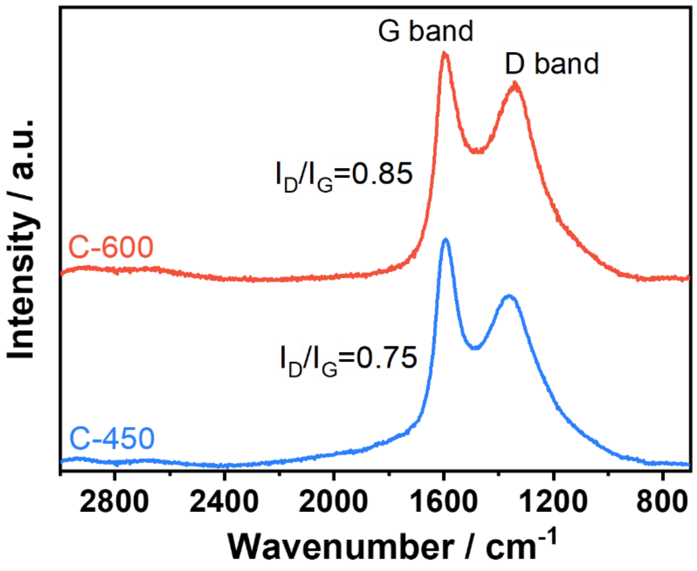

Figure S23. Raman spectra of C-450 and C-600.

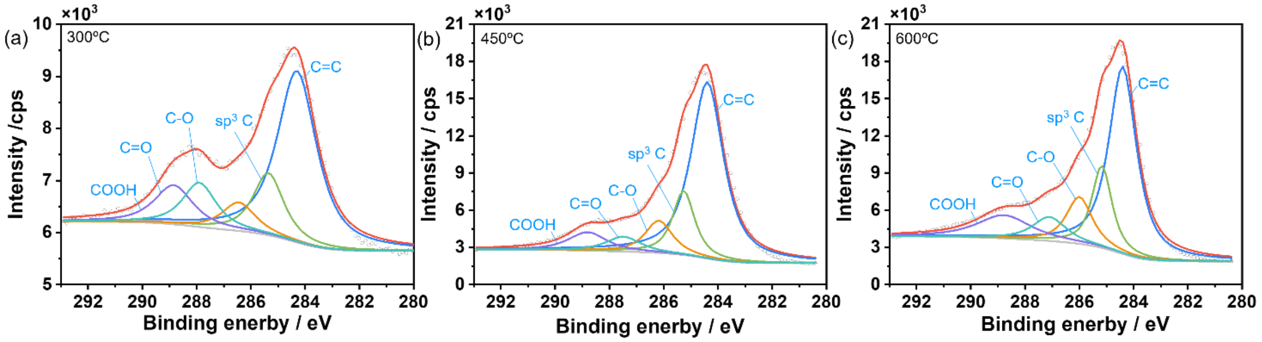

Figure S24. C 1s XPS spectra of carbon-coated CeO_2 composites obtained at CVD temperatures of (a) 300 °C, (b) 450 °C, and (c) 600 °C.

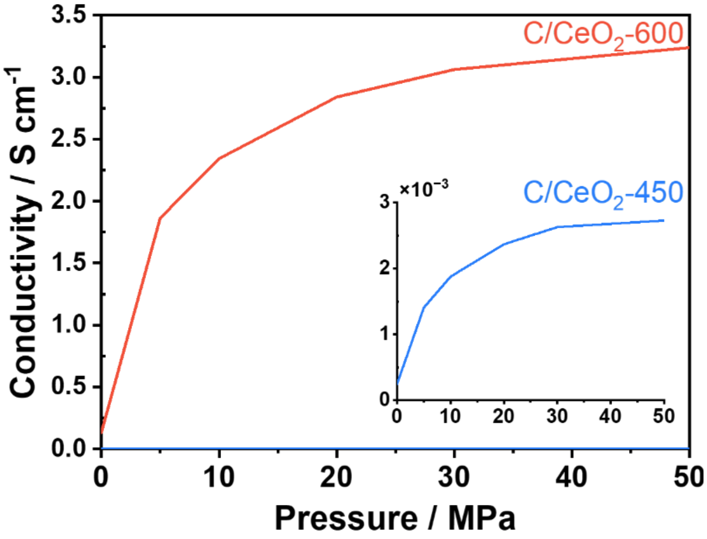

Figure S25. Electrical conductivity versus applied pressure for C/CeO_2-450 and C/CeO_2-600. The inset shows an enlarged view of the low-conductivity region for C/CeO_2-450.

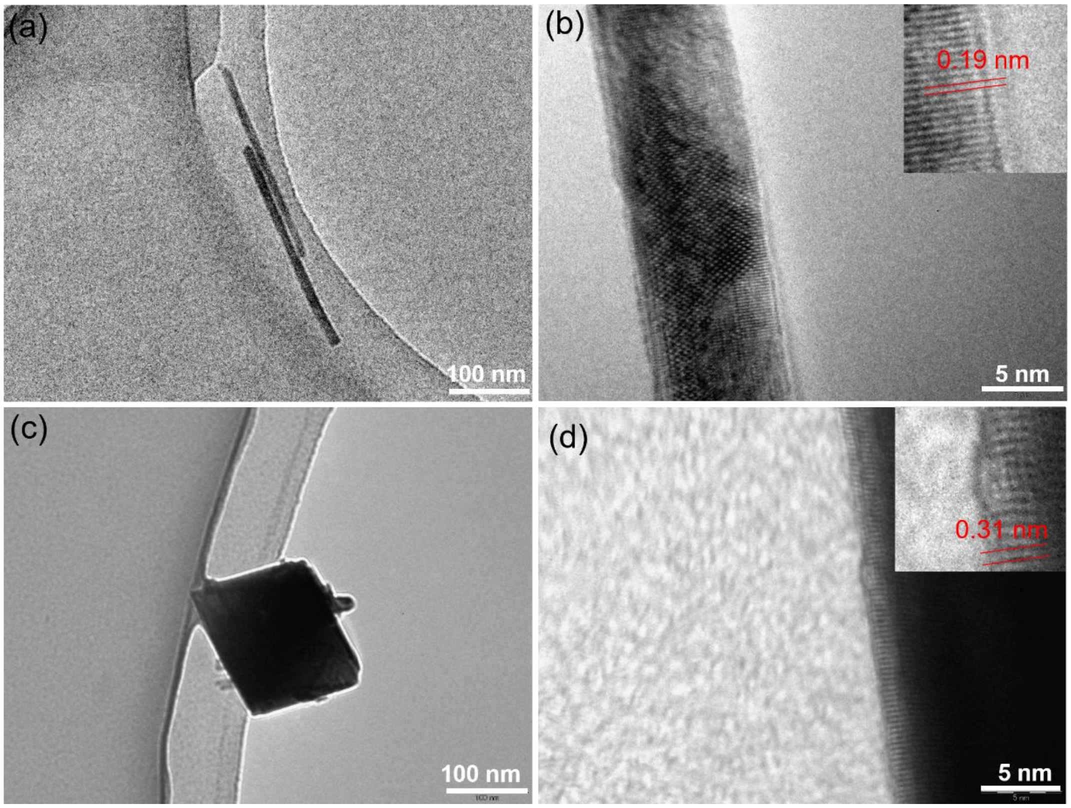

Figure S26. TEM images of (a, b) CeO_2-rod and (c, d) CeO_2-octa. The insets in (b) and (d) show enlarged views to clarify the lattice spacing.

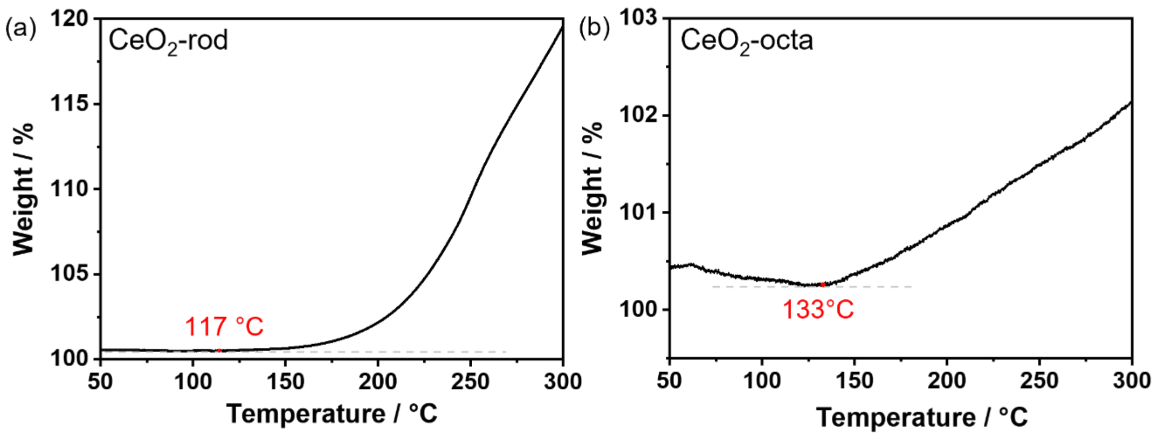

Figure S27. Weight change of (a) CeO_2-rod and (b) CeO_2-octa under a mixture gas flow of C_2H_2 (20 v%) and Ar monitored by TG.

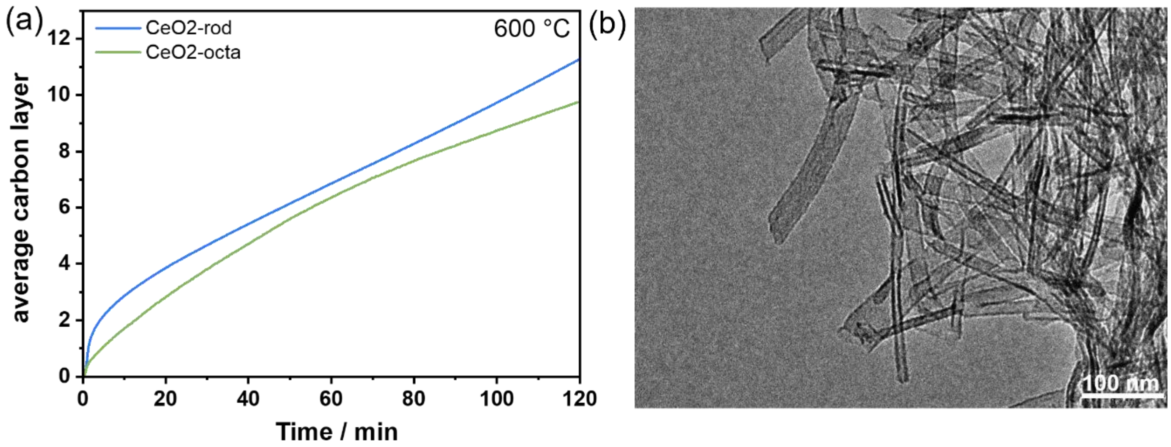

Figure S28. (a) TG-CVD curves of CeO_2-rod and CeO_2-octa at 600 °C. (b) TEM image of C-rod.

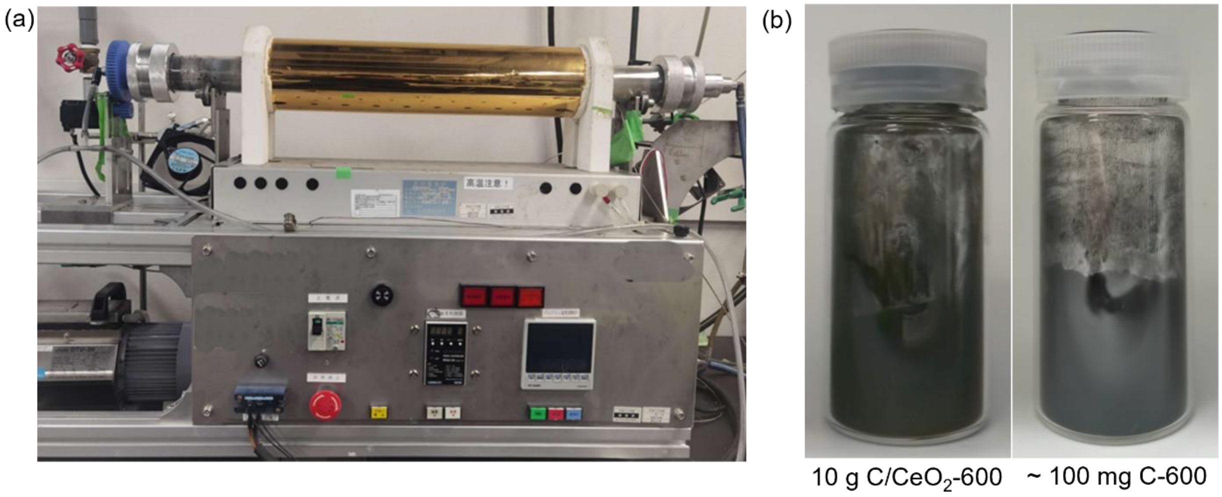

Figure S29. (a) Desktop rotary kiln furnace used for large-scale preparation. (b) Photographs of large-scale C/CeO_2-600 and the corresponding C-600 after template removal.

Table S1. Ce^{3+} ratio calculated by XPS data.

Position Area (eV)	sample name	CeO_2	CeO_2-HT-300C	CeO_2-HT-600C	C/CeO_2-150	C/CeO_2-300	C/CeO_2-450	C/CeO_2-600	C/CeO_2-300-10min
Ce^{4+}	u'''+v'''	2313.3	2361.7	1238.0	1143.5	899.0	996.9	1139.4	1113.5
	u''+v''	2287.2	2328.4	1252.9	1144.8	875.4	840.8	1210.6	928.7
	u^a+v^a	729.5	738.5	422.4	464.0	434.2	340.9	400.7	355.1
	u^b+v^b	1593.8	1586.5	907.4	996.7	932.7	732.3	860.8	762.9
Ce^{3+}	u^0+v^0	0.1	0.1	0.2	0.1	204.6	109.8	0.1	208.2
	u'+v'	701.5	725.3	536.1	610.2	927.5	427.3	480.4	596.2
	$A(Ce^{3+}+Ce^{4+})$	7625.4	7740.6	4357.1	4359.3	4273.3	3447.9	4092.0	3964.7
	$A(Ce^{3+})$	701.6	725.4	536.3	610.3	1132.1	537.0	480.5	804.4
	C_{Ce3+}	9.2	9.4	12.3	14.0	26.5	15.6	11.7	20.3

Table S2. The comparison of oxygen vacancy formation energies on MgO and CeO_2 systems.

	MgO(110)-Vo	C_2H_2-MgO(110)-Vo	CeO_2(110)-Vo	C_2H_2-CeO_2(110)-Vo
Evo / eV	4.95	4.96	1.46	1.31
